# Recent Advancement in Fluorescent Probes for Peroxynitrite (ONOO^−^)

**DOI:** 10.3390/s25103018

**Published:** 2025-05-10

**Authors:** Hai-Hao Han, Pan-Xin Ge, Wen-Jia Li, Xi-Le Hu, Xiao-Peng He

**Affiliations:** 1 Key Laboratory for Advanced Materials and Joint International Research, School of Chemistry and Molecular Engineering, East China University of Science and Technology, 130 Meilong Rd., Shanghai 200237, China; hanhaihao@simm.ac.cn (H.-H.H.); xlhu@ecust.edu.cn (X.-L.H.); 2 Laboratory of Precision Chemistry and Molecular Engineering, School of Chemistry and Molecular Engineering, East China University of Science and Technology, 130 Meilong Rd., Shanghai 200237, China; 3 Feringa Nobel Prize Scientist Joint Research Center, School of Chemistry and Molecular Engineering, East China University of Science and Technology, 130 Meilong Rd., Shanghai 200237, China; 4 Frontiers Center for Materiobiology and Dynamic Chemistry, School of Chemistry and Molecular Engineering, East China University of Science and Technology, 130 Meilong Rd., Shanghai 200237, China; 5 Shandong Laboratory of Yantai Drug Discovery, Bohai Rim Advanced Research Institute for Drug Discovery, Yantai 264117, China; 2023254227027@stu.ahtcm.edu.cn (P.-X.G.); liwenjia@simm.ac.cn (W.-J.L.); 6 Molecular Imaging Center, National Center for Drug Screening, Stake Key Laboratory of Chemical Biology, Shanghai Institute of Materia Medica, Chinese Academy of Sciences, Shanghai 201203, China; 7 University of Chinese Academy of Sciences, No. 19A Yuquan Road, Beijing 100049, China; 8 School of Pharmacy, Anhui University of Chinese Medicine, Hefei 230031, China; 9 The International Cooperation Laboratory on Signal Transduction, National Center for Liver Cancer, Eastern Hepatobiliary Surgery Hospital, Shanghai 200438, China

**Keywords:** peroxynitrite (ONOO^−^), fluorescent probes, bioimaging, in vivo, disease diagnosis and treatment

## Abstract

Peroxynitrite (ONOO^−^) is a reactive nitrogen species (RNS) that plays pivotal roles in various physiological and pathological processes. The recent literature has seen significant progress in the development of highly sensitive and selective fluorescent probes applicable for monitoring ONOO^−^ dynamics in live cells and a variety of animal models of human diseases. However, the clinical applications of those probes remain much less explored. This review delves into the biological roles of ONOO^−^ and summarizes the design strategies, sensing mechanisms, and bioimaging applications of near-infrared (NIR), long-wavelength, two-photon, and ratiometric fluorescent probes modified with a diverse range of functional groups responsive to ONOO^−^. Furthermore, we will discuss the remaining problems that prevent the currently developed ONOO^−^ probes from translating into clinical practice.

## 1. Introduction

Peroxynitrite (ONOO^−^), a potent oxidizing agent formed via the reaction between nitric oxide (•NO) and superoxide anion (O_2_^•−^), is integral to oxidative stress, cellular damage, and the pathogenesis of various diseases in organisms [1,2]. ONOO^−^ participates in numerous biological processes including cell signaling, inflammation, and apoptosis [3,4]. In the context of oxidative stress, ONOO^−^ functions in a concentration-dependent way. At moderate concentrations, ONOO^−^ acts as a signaling molecule fostering physiological functions; however, an excessive level of ONOO^−^ can damage DNA, proteins, and lipids, thereby resulting in severe health issues such as hepatotoxicity, cardiovascular diseases, neurodegenerative disorders, and cancer [5,6]. Consequently, elucidating the biological functions of ONOO^−^ and its relevance to human diseases is an important research topic. By monitoring fluctuations in ONOO^−^ concentration, researchers can gain insights into cellular oxidation states and the corresponding physiological responses, facilitating the identification of potential disease biomarkers [7,8]. In addition, the accurate measurement of ONOO^−^ levels can improve the management of oxidative-stress-associated diseases [9,10].

Traditional analytical methods for ONOO^−^ include ultraviolet–visible (UV–vis) spectrometric, electrochemical, and colorimetric assays, which require complicated sample processing and analyses and are not suitable for monitoring ONOO^−^ in vivo [11,12,13]. Comparing to conventional imaging modes such as magnetic resonance imaging and computed tomography, which suffer from issues like high cost and low sensitivity [14,15], fluorescence-based techniques are advantageous in terms of sensitivity, selectivity, as well as imaging precision. A diverse range of fluorescence probes have been developed in the past decade for the selective detection of ONOO^−^ [16,17,18]. In recent years, several pertinent reviews have already been published. Liu et al. summarized ONOO^−^ probes based on structurally different fluorophores [10]. Ma et al. reviewed small-molecule ratiometric fluorescent probes for ONOO^−^ detection from 2017 to 2022 [19]. Yang et al. summarized diagnostic probes for sensing ONOO^−^ in different disease models [20]. Li et al. summarized organelle-targeted fluorescent probes for ONOO^−^ detection [16]. Grzelakowska et al. reviewed molecular probes based on boronates and carbonyl groups for ONOO^−^ detection [21]. Li et al. summarized fluorescent probes for the simultaneous detection of ONOO^−^ and other analytes [22]. Zhou et al. systematically reviewed the development of fluorescent probes for the single and dual detection of three reactive oxygen species (superoxide anion, nitric oxide, and ONOO^−^) from 2020 to 2023 [23]. While these reviews offer valuable insights into the development of chemical tools for ONOO^−^ detection, a comprehensive analysis of the design mechanisms and applications of ONOO^−^-responsive probes remains elusive. In addition, the present review also includes representative fluorescent probes developed since 2024 for ONOO^−^ sensing and imaging in vivo.

**Figure 1:**
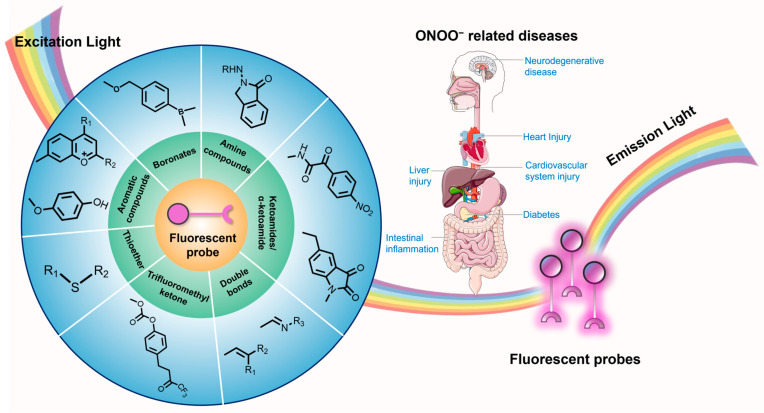
Schematic illustration of previous fluorescent probes developed for the sensing of ONOO^−^ in vivo.

We comprehensively summarize the current design strategies of near-infrared (NIR), two-photon, and ratiometric fluorescent probes for the detection of ONOO^−^. The sensing mechanisms of the probes based on different functional groups responsive to ONOO^−^, and the applications of the probes for dynamically tracking the expression level of ONOO^−^ in various animal models will be discussed (Figure 1). Additionally, the remaining challenges associated with the currently developed fluorescent probes for ONOO^−^ sensing and clinical translation will be proposed.

## 2. ONOO^−^

### 2.1. Generation of ONOO^−^

**Figure 2:**
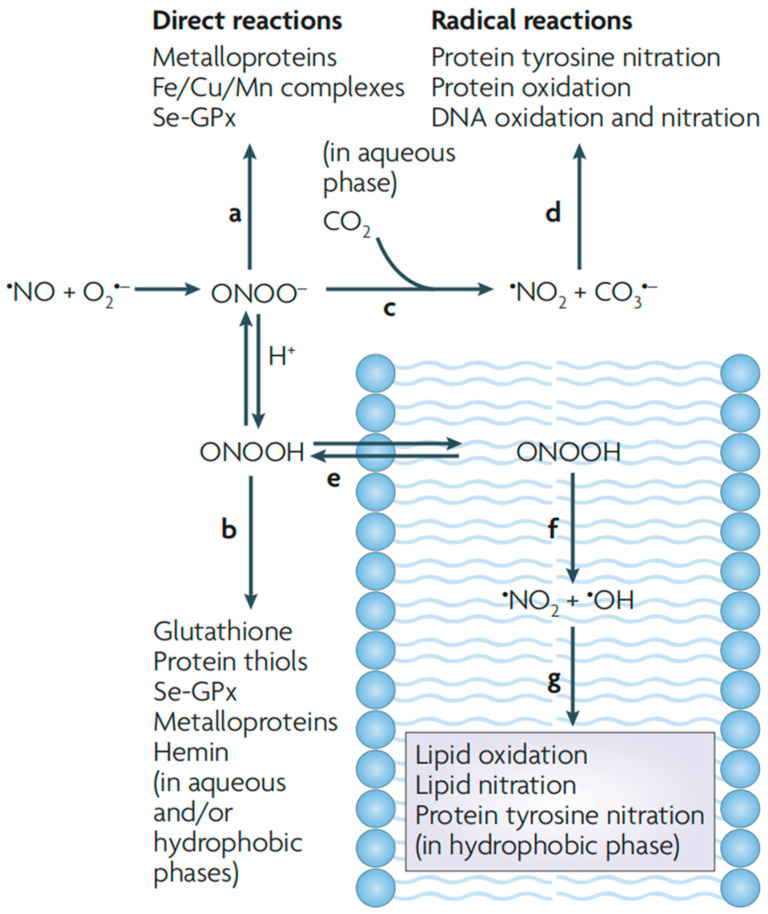
Generation and biological roles of ONOO^−^. Reproduced with permission from [8].

ONOO^−^ is a short-lived oxidant species produced by the reaction of •NO with O_2_^•−^ (Figure 2) [8]. Under physiological conditions, the productivity of ONOO^−^ is relatively low, and oxidative damage is minimized owing to the body’s endogenous antioxidant defense mechanisms [24]. Under pathological conditions, the concentration of ONOO^−^ is significantly increased, leading to the oxidation and destruction of cellular components. This disruption can result in the dysfunction of key metabolic processes of cells and even trigger cell death via apoptosis or necrosis [3]. Consequently, ONOO^−^ is an important target for the study of disease biology.

### 2.2. ONOO^−^ and Diseases

ONOO^−^ can interact with lipids [25], DNA [26], and proteins [27] via direct oxidation or indirect free-radical-mediated mechanisms. These interactions may induce oxidative stress, activate inflammatory signaling pathways such as those mediated by NF-κB [28], and disrupt cellular signal-transduction processes [29], leading to mitochondrial dysfunction and apoptosis [30]. The abnormal accumulation of ONOO^−^ is implicated in diseases including myocardial infarction [31,32], chronic heart failure [33], diabetes [34], chronic inflammatory diseases [35], cancer [36], neurodegenerative diseases [37], and liver diseases [38]. As a consequence, the effective monitoring of ONOO^−^ fluctuation could become a promising approach for disease diagnosis and assessment of the efficacy of antioxidant therapies.

### 2.3. ONOO^−^ Detection

Conventional analytical methods for ONOO^−^ include spectrometry (e.g., UV–vis spectrometry), chromatography (e.g., high-performance liquid chromatography, HPLC), and enzyme-linked immunosorbent assay (ELISA), among others. However, all of these methods have drawbacks. Spectrometry, which relies on chromogenic reactions, has relatively low sensitivity (at the μM level) and is easily impacted by other reactive oxygen species (ROS) such as H_2_O_2_ and ClO^−^ [39]. While HPLC is sensitive enough for ONOO^−^ detection, it necessitates complicated pre-treatment procedures and indirectly measures the analyte through the detection of nitrification products, thereby precluding the real-time monitoring of ONOO^−^ dynamics [40]. ELISA, which employs specific antibodies to capture ONOO^−^, is susceptible to cross-reactions [41].

In recent years, fluorescent probes have been extensively developed for ONOO^−^. Fluorescent probes offer high sensitivity with LoDs reaching the nanomolar level, as well as high selectivity for ONOO^−^ when the responsive group for the RNS is exquisitely designed [42]. Small-molecule probes can also be used directly for cell-based and in vivo fluorescence imaging, thus making them suitable for real-time monitoring of the generation of ONOO^−^ during various disease-relevant processes [43]. The development of these probes facilitates both chemical biological research of ONOO^−^ and the validation of its potential for disease diagnosis. In the following section, we will summarize the recently developed fluorescent probes for ONOO^−^ based on different sensing mechanisms and discuss their biological applications.

## 3. Fluorescent Probes for Detection of ONOO^−^

### 3.1. Fluorescent Probes Based on Boronates (Boronic Acids/Boronic Esters)

Boron exhibits an sp^2^ hybridization with an empty p orbital, resulting in a triangular plane configuration that confers significant Lewis acidity to boron-containing compounds [44]. This chemical property makes boronates highly reactive with nucleophiles. Boron-containing compounds have shown susceptibility to nucleophilic attack by substances such as H_2_O_2_, leading to structural alterations [45]. Recent research has indicated that ONOO^−^, due to its strong nucleophilicity, could react with boronic acids more efficiently than other ROS like H_2_O_2_ [46]. This distinctive reactivity makes boron-containing compounds an ideal candidate for ONOO^−^ sensing, and, therefore, fluorescent probes based on boronates have been developed.

**Figure 3:**
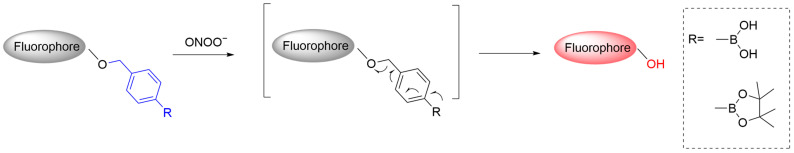
Schematic illustration of the sensing mechanism of boronate-based fluorescent probes for ONOO^−^.

In recent years, boronic acids and esters, as boron-containing organic compounds, have been exploited for the development of ONOO^−^ fluorescent probes [47,48]. Boronates are directly coupled to a fluorophore, and in some cases arylboronic acids are used to improve sensitivity and selectivity (Figure 3) [49,50]. Boron-containing fluorescent probes for ONOO^−^ have proved effective not only in identifying and quantifying ONOO^−^ but providing powerful tools for the in-depth investigation of its mechanism of action in biological systems [51].

**Figure 4:**
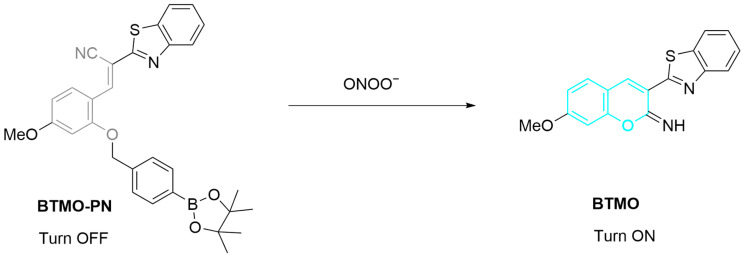
Schematic illustration of the sensing mechanism of probe **BTMO-PN** for ONOO^−^.

Ma et al. developed an ONOO^−^ probe, **BTMO-PN**, through coupling phenylboronic ester to a benzothiazolyl derivative, for monitoring ONOO^−^ generation during ferroptosis (Figure 4) [52]. The strong nucleophilicity of ONOO^−^ induces the cleavage of the boronic ester, forming a phenolic hydroxyl intermediate, which then undergoes rapid intramolecular cyclization to produce a fluorescent species, benzothiazoly-iminocoumarin (**BTMO**). Upon reaction with ONOO^−^, the fluorescence of **BTMO-PN** (emission maximum (λ_em-max_) at 477 nm) is significantly enhanced. The probe was used for ONOO^−^ detection in HeLa (human cervical) cells after treatment with different agents including lipopolysaccharide (**LPS**), interferon-γ (**IFN-γ**), and phorbol 12-myristate 13-acetate (**PMA**) that upregulate ONOO^−^ concentration in cells. Notably, a strong fluorescence was detected in a ferroptosis model constructed by treating HeLa cells with erastin, suggesting that ONOO^−^ levels are upregulated during ferroptosis. In a KM mouse model injected subcutaneously with H22 (mouse hepatoma) cells, **BTMO-PN** was used to distinguish normal tissues from tumor tissue, with the fluorescence signal in the latter being ~3.5 times stronger than that in the former, suggesting a high expression of ONOO^−^ in tumor cells. This study provides an effective imaging tool for investigating ONOO^−^-mediated cell ferroptosis.

**Figure 5:**
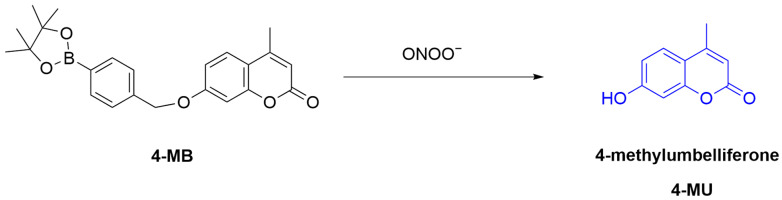
Schematic illustration of the sensing mechanism of probe **4-MB** for ONOO^−^.

Ratiometric fluorescent probes quantitatively measure target analytes through changes in fluorescence ratios [53]. These probes typically combine different fluorescence emission wavelengths, which, through ratiometric analysis, mitigates the impact of environmental factors, thereby enhancing the accuracy and sensitivity of measurements [54]. Ratiometric fluorescent probes have demonstrated excellent selectivity and sensitivity in the detection of ONOO^−^. Palanisamy et al. developed a ratiometric fluorescent probe, **4-MB** (Figure 5) [55]. The sensing mechanism is the oxidation of aryl boronate by ONOO^−^, resulting in the conversion of the probe to 4-methylumbelliferone. This conversion red-shifts the λ_em-max_ of the probe from 385 nm to 450 nm, thus enabling fluorescence-based ratiometric analyses by referring to the fluorescence intensity ratio (λ_450_/λ_385_) changes after reaction with ONOO^−^. The probe was successfully used for the generation of ONOO^−^ in RAW264.7 (murine macrophages) and EAhy926 (human umbilical vein endothelial) cells, after treatment with **LPS**, **IFN-γ**, or **PMA**. Next, the probe was administrated to a high-fat-diet-induced C57BL/6J mouse model, revealing a significantly increased ONOO^−^ level in the kidneys, suggesting that high-fat diet upregulated ONOO^−^ could lead to kidney damage.

**Figure 6:**
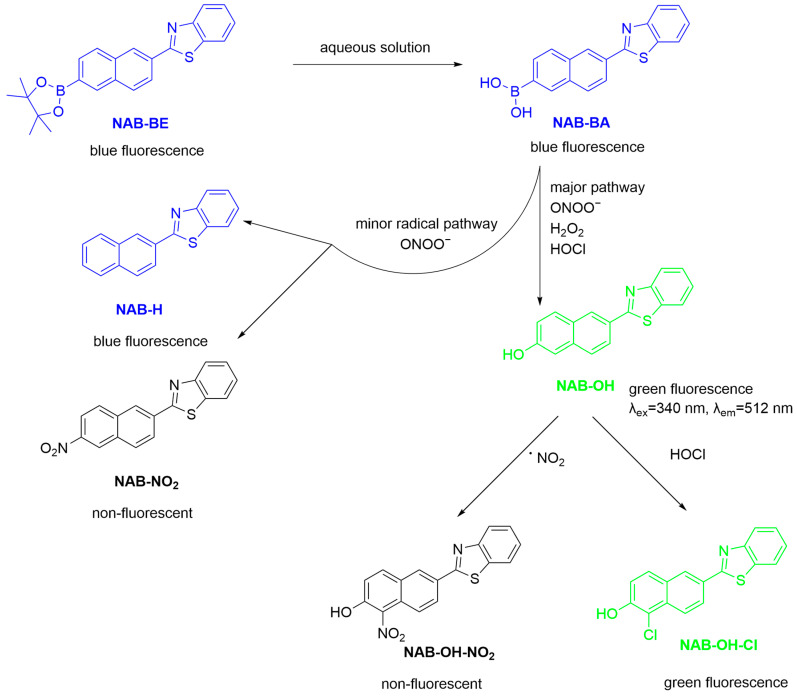
Schematic illustration of the sensing mechanism of the probe **NAB-BE** for ONOO^−^.

Two-photon fluorescent probes are highly sensitive bioimaging tools that utilize two-photon excitation to detect biomolecules in live cells and animals [56]. This technique employs NIR light sources, which can penetrate deeply into tissues, thus reducing phototoxicity and enhancing spatial resolution. Typically, two-photon probes exhibit low background fluorescence, resulting in superior signal-to-noise ratios for in situ imaging [57]. Grzelakowska et al. developed a two-photon fluorescent probe, **NAB-BE**, for the sensitive detection of ONOO^−^ (Figure 6) [58]. The probe reacts with inflammatory oxidants including ONOO^−^, HOCl, and H_2_O_2_, producing a strongly green-fluorescent phenolic product, **NAB-OH**. Notably, ONOO^−^ reacts with the probe ~1 million times faster than H_2_O_2_ and >30 times faster than HOCl. The naphthalene and benzothiazoly groups of the probe form an efficient electron donor-π-acceptor structure, facilitating intramolecular charge transfer (ICT) to enhance fluorescence. The probe is compatible with two-photon fluorescence microscopy (λ_Ex/Em_ = 745 nm/465–550 nm) and achieved imaging of ONOO^−^ generation in RAW 264.7 macrophages stimulated by **LPS**, **IFN-γ**, and **PMA**. Given its compatibility with two-photon excitation, the authors envisioned the application of the probe for in vivo imaging.

**Figure 7:**
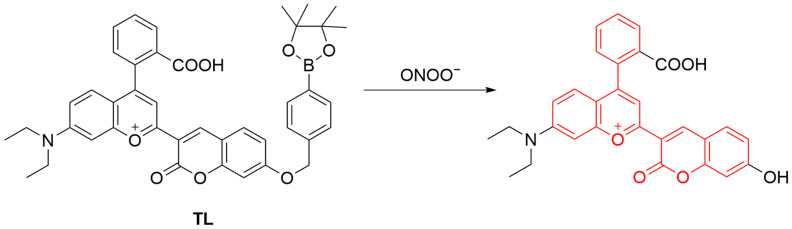
Schematic illustration of the sensing mechanism of the probe **TL** for ONOO^−^.

Long-wavelength fluorescent probes are advantageous in terms of imaging deep tissues, minimizing photodamage to biological samples, reducing background auto-fluorescence, and enhancing the imaging signal-to-noise ratio [59]. Tang et al. developed a long-wavelength mitochondrial-targeting fluorescent probe, **TL**, for the detection of ONOO^−^ (Figure 7) [60]. The fluorescence of the probe is “turned on” through reaction between its phenylboronic acid group and ONOO^−^, resulting in the formation of a primary phenolic product with a λ_em-max_ at 667 nm. The probe was successfully applied for ONOO^−^ imaging in HepG2 (human hepatoma) cells. The positive charge of **TL** facilitates its interaction with the negatively charged mitochondrial membrane. Furthermore, the generation of ONOO^−^ in cells stimulated with dexamethasone was visualized by the probe.

**Figure 8:**
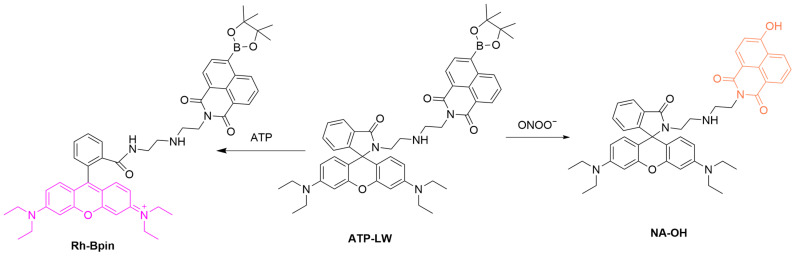
Schematic illustration of the sensing mechanism of the probe **ATP-LW** for ONOO^−^ and ATP.

Dual-channel fluorescent probes are designed to detect two analytes simultaneously [61]. Through utilizing spectrally well-separated excitation and emission wavelengths, they can be used to monitor the dynamic changes of multiple biomolecules within a single test sample. Our group developed a two-channel fluorescent probe, **ATP-LW**, which can detect both ONOO^−^ and ATP (Figure 8) [62]. When **ATP-LW** reacts with ONOO^−^, the boronic ester component of the probe is partially oxidized, resulting in the formation of a fluorescence-enhanced species, **NA-OH** (λ_ex_/λ_em_ = 450 or 488 nm/562 or 568 nm). In contrast, when ATP binds to the probe, it induces the opening of the spirolactam ring in the rhodamine fluorophore (λ_ex_/λ_em_ = 520 nm/587 nm), producing a strong fluorescence in a different channel from that used for ONOO^−^ detection. **ATP-LW** was successfully used for the simultaneous detection of ONOO^−^ and ATP in HL-7702 (human hepatocytes) cells pretreated with acetaminophen (**APAP**), which induces liver cell injury. The imaging results suggest an upregulated and downregulated level of ONOO^−^ and ATP upon drug treatment, respectively, thus offering a powerful tool for the study of disease biology.

**Figure 9:**
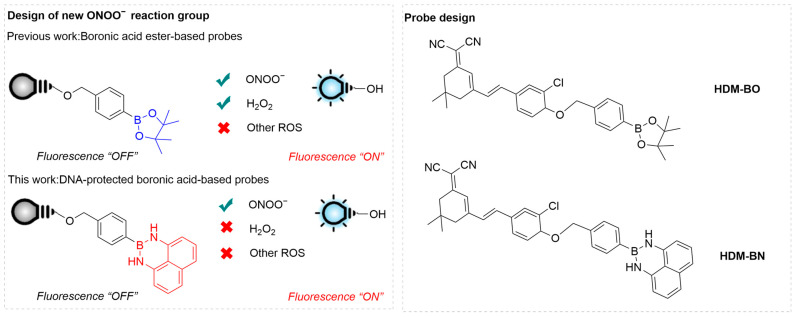
Design principle for the **DAN**-protected boronic-acid-based probes and structural design of the probe **HDM-BN**. Reproduced with permission from [63].

Gong et al. employed diaminonaphthalene-protected boronic acid (**DANBA**) as a new responsive group for ONOO^−^ to couple with (E)-2-(3-(3-chloro-4-hydroxystyryl) cyclohex-2-en-1-ylidene) malononitrile (**HDM**), forming a D-A-type conjugate **HDM-BN** (Figure 9) [63]. Upon reaction with ONOO^−^, the weak donor (alkoxy group) in **DANBA** is converted to a stronger donor (hydroxyl group), thereby enhancing the ICT, and thus the fluorescence, of the conjugate. This unique reaction mechanism allows **HDM-BN** to selectively recognize ONOO^−^ over other ROS. In an aqueous solution, the λ_ex_ of **HDM-BN** red-shifts from 425 nm to 530 nm after reaction with ONOO^−^ along with a significantly enhanced fluorescence at λ_em-max_ of 689 nm. **HDM-BN** effectively monitored added and simulated ONOO^−^ in PC12 (murine pheochromocytoma) cells. The produced fluorescence intensity positively correlated with the ONOO^−^ levels after treatment with various concentrations of 3-morpholinosydonimine hydrochloride (**SIN-1**). Using a mouse model of Parkinson’s disease induced by 1-methyl-4-phenyl-1,2,3,6-tetrahydropyridine (**MPTP**), **HDM-BN** achieved real-time imaging of the mouse brain to monitor dynamic changes in ONOO^−^ levels. The results revealed a significantly higher fluorescence signal in the **MPTP**-treated group compared to that in the control group.

**Figure 10:**
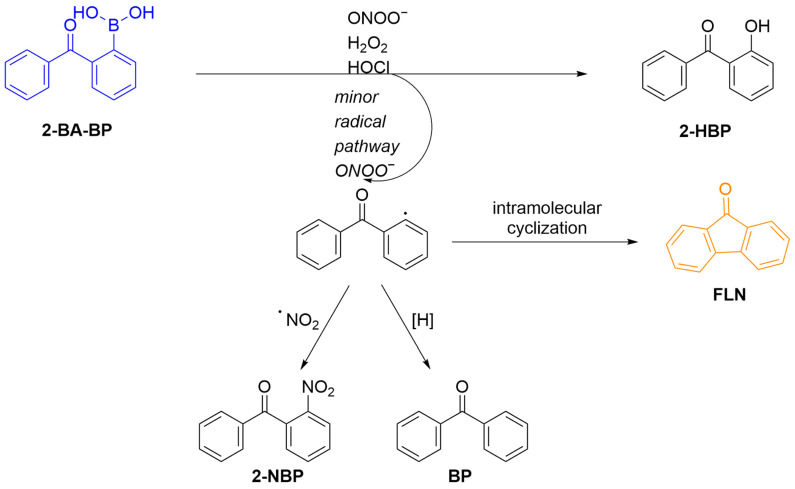
Schematic illustration of the sensing mechanism of the probe **2-BA-BP** for ONOO^−^.

Grzelakowska et al. developed a new boronate probe (**2-BA-BP**) for selectively detecting ONOO^−^ (Figure 10) [64]. This probe generates a fluorescent product, **FLN**, through an intramolecular cyclization reaction. **FLN** exhibits strong green fluorescence at a maximum emission wavelength of 500 nm upon excitation at 256 nm. The probe **2-BA-BP** effectively monitors the formation of ONOO^−^ induced by **SIN-1** in situ, suggesting the potential of employing boronate probes for ONOO^−^ analysis in biological systems.

### 3.2. Fluorescent Probes Based on Hydrazides

**Figure 11:**
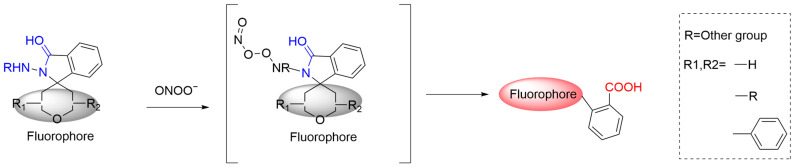
Schematic illustration of the reaction mechanism between hydrazide-based fluorescent probes and ONOO^−^.

Hydrazides can react with ONOO^−^ to produce stable nitrogen oxides. The incorporation of hydrazide into the spiro-ring system of rhodamine is a popular strategy for designing fluorescent probes [65]. Upon reaction with ONOO^−^, the hydrazide group of the probe is converted to a carboxylic group leading to the opening of the spirolactone ring of rhodamine, thus giving rise to fluorescence (Figure 11) [66].

**Figure 12:**
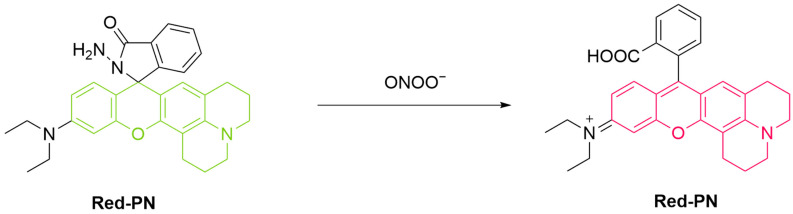
Schematic illustration of the sensing mechanism of the probe **Red-PN** for ONOO^−^.

Li et al. developed a red-emitting fluorescent probe, **Red-PN**, for the detection of ONOO^−^ (Figure 12) [67]. The probe is a hydrazide-modified rhodamine dye that forms a non-fluorescent spirocyclic structure. Upon reaction with ONOO^−^, the spirocyclic ring opens, leading to fluorescence recovery. This reaction occurs within 5 s, which allows the probe to detect low concentrations of ONOO^−^ over a short period (limit of detection (LoD) = 4.3 nM). **Red-PN** achieved fluorescence imaging of RAW 264.7 macrophages as well as zebrafish treated with ONOO^−^, **PMA**, or **LPS**, suggesting its usefulness for ONOO^−^ monitoring both in live cells and in vivo.

**Figure 13:**
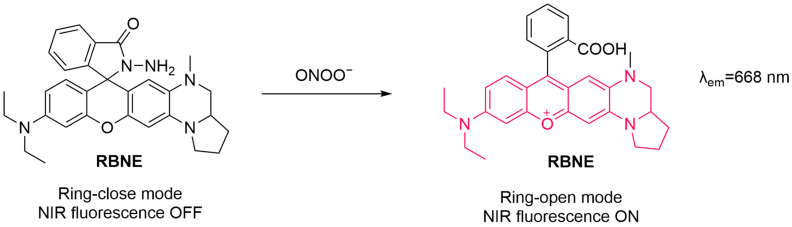
Schematic illustration of the sensing mechanism of the probe **RBNE** for ONOO^−^.

Similarly, Zhang et al. developed an NIR fluorescent probe, **RBNE**, for the sensitive detection and imaging of ONOO^−^ in necrotizing enteritis (NEC) (Figure 13) [68]. The probe sensitively detects ONOO^−^ with an LoD of 56 nM. The fluorescence of **RBNE** was markedly enhanced in HepG2 cells after treatment with **SIN-1**. In an NEC mouse model, **RBNE** was employed to monitor ONOO^−^ levels, revealing fluorescence in the abdominal region of mice. This study offers a chemical tool that could potentially become useful for the diagnosis of NEC.

**Figure 14:**
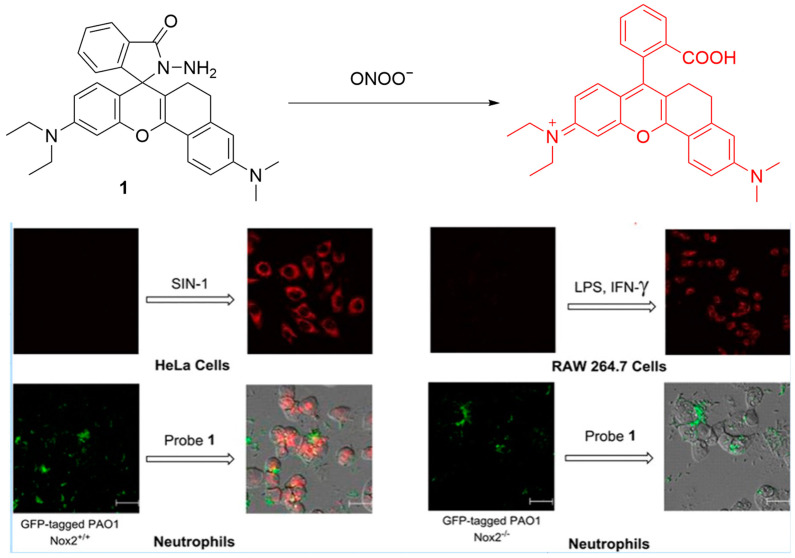
Schematic illustration of the sensing mechanism of **Probe 1** for ONOO^−^. Reproduced with permission from [69].

Wu et al. developed a far-infrared emitting fluorescence probe, denoted as **Probe 1**, for the detection and imaging of ONOO^−^ (Figure 14) [69]. With a λ_ex_ of 600 nm and a λ_em-max_ of 638 nm, **Probe 1** demonstrates good sensitivity and selectivity for ONOO^−^. It responds linearly to ONOO^−^ concentrations ranging from 0 to 34 μM, with an LoD of 45 nM. In HeLa cells, the fluorescence of **Probe 1** was significantly enhanced upon the addition of **SIN-1**. In addition, ONOO^−^ generated by **LPS**- and **IFN-γ**-pretreated RAW 264.7 macrophages could also be visualized by the probe. In vivo imaging using **Probe 1** indicated an increase in ONOO^−^ concentration in neutrophils isolated from the bronchoalveolar lavage (BAL) fluid of wild-type mice infected with *Pseudomonas aeruginosa* (PAO1). However, minimal ONOO^−^ signal was detected in Nox2 (NADPH oxidase 2)-deficient mice infected with PAO1, suggesting that ONOO^−^ is upregulated in neutrophils from BAL fluids of PAO1-infected wild-type mice, and that a deficiency in Nox2 substantially suppresses the ONOO^−^ level.

**Figure 15:**
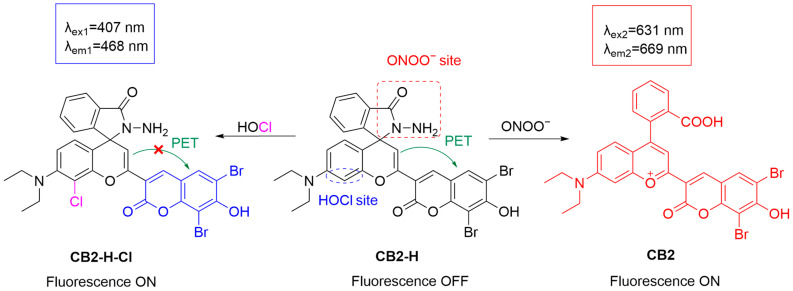
Schematic illustration of the proposed sensing mechanism of **CB2-H** for the simultaneous detection of HOCl and ONOO^−^.

In addition to detecting just ONOO^−^, dual-channel systems have also been developed for the simultaneous detection of ONOO^−^ and another analyte. Huang et al. developed a dual-channel fluorescent probe, **CB2-H**, capable of simultaneously detecting ONOO^−^ and HOCl in biological systems (Figure 15) [70]. Upon reaction with ONOO^−^, the hydrazide moiety in **CB2-H** is selectively oxidized, leading to the opening of the spirolactam ring and the formation of the parent dye **CB2**, which has a λ_em-max_ of 669 by excitation at 631 nm. In contrast, HOCl electrophilically attacks the benzopyran component of the probe, producing a chlorinated product, **CB2-H-Cl**. This product inhibits the photo-induced electron transfer (PeT) process of the probe, resulting in a strong fluorescence signal with a λ_em-max_ of 468 nm by excitation at 407 nm. Fluorescence imaging experiments using **CB2-H** were conducted on HeLa cells and RAW 264.7 cells treated with different stimuli. The results demonstrated that the fluorescence signal in the blue channel was significantly enhanced in cells treated with NaOCl, while the red-channel signal was significantly enhanced upon the addition of ONOO^−^. Furthermore, experiments using inhibitors including aminoguanidine (an inhibitor of nitric oxide synthase suppressing the formation of ONOO^−^) and 4-aminobenzoic acid hydrazide (an inhibitor of myeloperoxidase that reduces the generation of HOCl) validated the selectivity of the probe. In a zebrafish model, **CB2-H** was used to detect the generation of HOCl and ONOO^−^ induced by **APAP**. Following **APAP** treatment, fluorescence in both the blue and red channels was detected in the liver of zebrafish, demonstrating the imaging capacity of the probe **CB2-H** in vivo.

Lysosomes are crucial membranous organelles in cells, which are responsible for the degradation and recycling of cellular substances [71]. They contain a variety of hydrolases that break down large molecules such as proteins, lipids, nucleic acids, and polysaccharides, and are involved in cell metabolism and autophagy [72]. Lysosomes play a key role in maintaining intracellular homeostasis, removing damaged cellular components, and participating in cell signal transduction and immune responses [73]. Research has demonstrated that ONOO^−^ can induce lysosome-mediated cell death, underscoring the significance of detecting ONOO^−^ for study of lysosomal biology [16].

**Figure 16:**
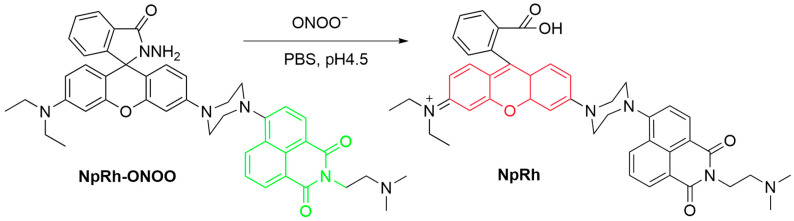
Schematic illustration of the sensing mechanism of the probe **NpRh-ONOO** for ONOO^−^.

Sun et al. synthesized a two-photon lysosomal-targeting fluorescent probe, **NpRh-ONOO**, for the detection of ONOO^−^ in biological systems (Figure 16) [74]. Upon reaction of the hydrazide moiety of **NpRh-ONOO** with ONOO^−^, the spirolactam ring of the probe opens, releasing the parent fluorescent dye **NpRh** and with a red-shifted fluorescence from λ_em-max_ of 505 nm to 578 nm. With an excitation wavelength of 780 nm, the probe allows for the quantitative detection of ONOO^−^ in a ratiometric manner. A dimethylamino group is introduced to the probe to target the lysosomes. Fluorescence imaging of HeLa cells revealed that the probe exhibited strong fluorescence in an emission channel of 495–530 nm, and upon stimulation with **LPS** and **IFN-γ**, the fluorescence in the original channel decreases, while that in a new channel of 550–580 nm increases simultaneously. The fluorescence of the probe in both channels is shown to be localized in the lysosomes. In a zebrafish model, **NpRh-ONOO** also effectively detected ONOO^−^ generation as simulated by the treatment of **LPS** and **IFN-γ**.

**Figure 17:**
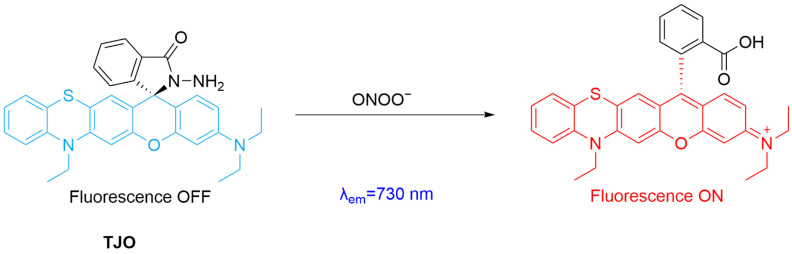
Schematic illustration of the sensing mechanism of the probe **TJO** for ONOO^−^.

Shang et al. developed a new long-wavelength fluorescent probe, **TJO**, for real-time monitoring of ONOO^−^ in cerebral ischemia–reperfusion injury (Figure 17) [75]. The probe is constructed through introducing hydrazine hydrate as an ONOO^−^-responsive group to a rhodamine derivative. Upon reacting with ONOO^−^, the hydrazine group is oxidized, leading to a significant fluorescence enhancement (at *λ*_em-max_ = 730 nm) as the result of the spiro-ring opening of the rhodamine derivative. SH-SY5Y cells pretreated with SIN-1 were used as a cell model for fluorescence imaging. In addition, a mouse middle artery obstruction model was established, and a more intensive fluorescence was seen in the reperfusion group than in the ischemia group, after treatment with **TJO**, indicating its effectiveness in detecting ONOO^−^ formation in ischemia–reperfusion injury.

### 3.3. Fluorescent Probes Based on Ketoamides

**Figure 18:**
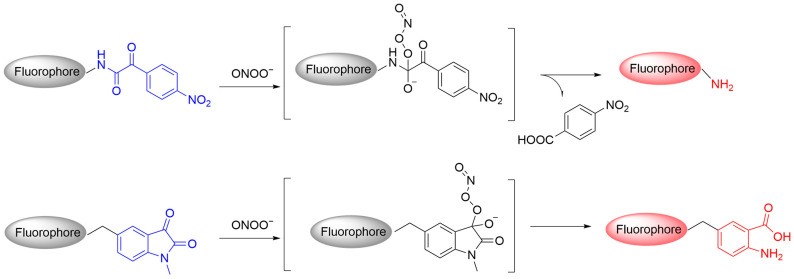
Schematic illustration of the reaction mechanism between α-ketoamide-based probes and ONOO^−^.

Ketoamides can be converted to ketones or amides after nucleophilic and redox reactions with ONOO^−^. Research has shown that linear α-ketoamide derivatives can be used as a responsive unit for ONOO^−^ in the design of fluorescent probes. Among these derivatives, 4-nitrobenzene-substituted α-ketoamide may initially undergo a nucleophilic attack by ONOO^−^, followed by a Baeyer–Villiger rearrangement and ultimately hydrolysis to yield an amine, 4-nitrobenzoic acid, and carbon dioxide (Figure 18) [76]. Isatin, a cyclic framework fused with an α-ketoamide group, reacts with ONOO^−^ after being coupled to a fluorophore. The attack on the ketone carbonyl group of isatin by ONOO^−^ leads to intramolecular cyclization and rearrangement, resulting in the formation of an anthranilic acid derivative and the release of the corresponding fluorophore through 1,6 elimination [77].

**Figure 19:**
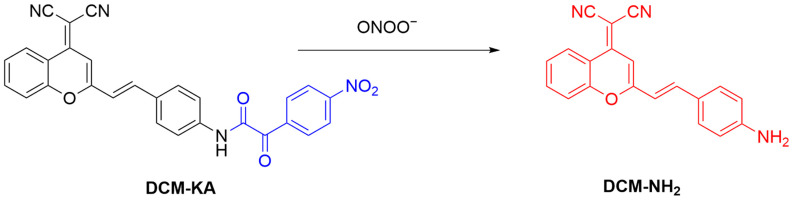
Proposed sensing mechanism of **DCM-KA** for ONOO^−^.

Qu et al. developed a far-infrared fluorescent probe, **DCM-KA**, designed to visualize intracellular ONOO^−^ production (Figure 19) [78]. The probe **DCM-KA** comprises an α-ketoamide and a **DCM-NH_2_** fluorophore. **DCM-KA** exhibits weak fluorescence in the absence of ONOO^−^; however, upon reaction with ONOO^−^, **DCM-NH_2_** is released, restoring the fluorescence signal. The probe has a λ_em-max_ of 630 nm, which falls within the far-infrared region, by excitation at 480 nm. **DCM-KA** was employed to image endogenous ONOO^−^ production in J774A.1 (murine macrophage) cells. Experimental results revealed a significantly enhanced fluorescence when cells are treated with **LPS** and **IFN-γ**, suggesting ONOO^−^ production. In a control experiment, the fluorescence of the probe was significantly diminished upon pretreatment of the cells with an ONOO^−^ inhibitor, aminoguanidine.

**Figure 20:**
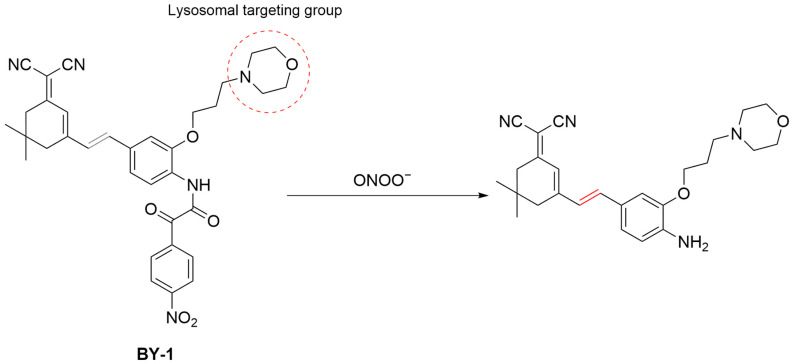
Schematic illustration of the sensing mechanism of the probe **BY-1** for ONOO^−^.

Li et al. developed **BY-1**, an NIR fluorescent probe for visualizing ONOO^−^ in osteoarthritis (OA) models (Figure 20) [79]. The probe consists of 4-nitrophenyl-2-oxoacetamide as an ONOO^−^-responsive group, dicyanoisophorone as fluorophore, and morpholine for lysosomal localization (λ_em-max_ of 668 nm). In TC28a2 (human chondrocytes) cells, **BY-1** successfully detected both exogenous (exposure to 30 μM ONOO^−^) and intracellularly generated ONOO^−^ (simulated by **IL-1β**), with its bright red fluorescence localized in the lysosomes. In an OA mouse model, subcutaneous injection of **BY-1** led to fluorescence production at the OA site over time, and the fluorescence decreased after treatment of therapeutic agents. **BY-1** might serve as a molecular tool for the diagnosis and therapeutic evaluation of OA.

**Figure 21:**
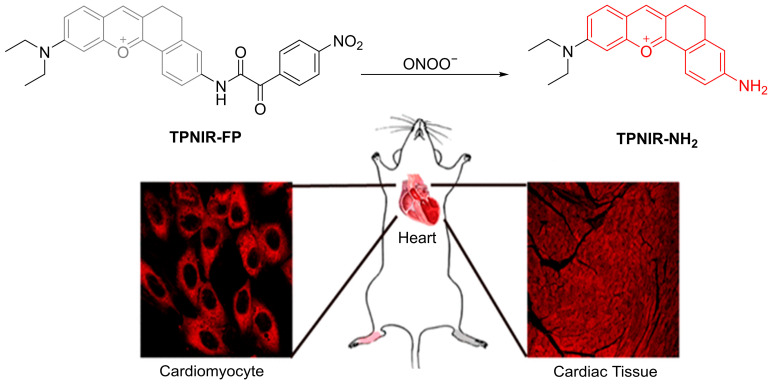
Schematic illustration of the detection of ONOO^−^ using **TPNIR-FP** ex vivo. Reproduced with permission from [80].

Xie et al. developed **TPNIR-FP**, a fluorescent probe for detecting ONOO^−^ in cardiomyocyte mitochondria, featuring two-photon excitation and NIR emission. (Figure 21) [80]. The probe, comprising **TPNIR-NH_2_** and an α-ketoamide bridge, targets mitochondria and fluoresces at λ_em-max_ of 630 nm upon reaction with ONOO^−^, with an LoD of 34 nM. In H9c2 (cardiac muscle) cells pretreated with **SIN-1**, **TPNIR-FP** showed a significantly enhanced red fluorescence and mitochondrial colocalization, indicating its ability to visualize intramitochondrial ONOO^−^. Dose-dependent fluorescence increases were observed in H9c2 cells stimulated with adriamycin and epirubicin, as well as in the heart sections of mice pretreated with the same agents following tail vein injection, demonstrating the probe’s effectiveness in visualizing anthracycline-induced cardiotoxicity. **TPNIR-FP** offers a potential diagnostic tool for early detection and treatment of cardiotoxicity associated with anthracycline medications.

**Figure 22:**
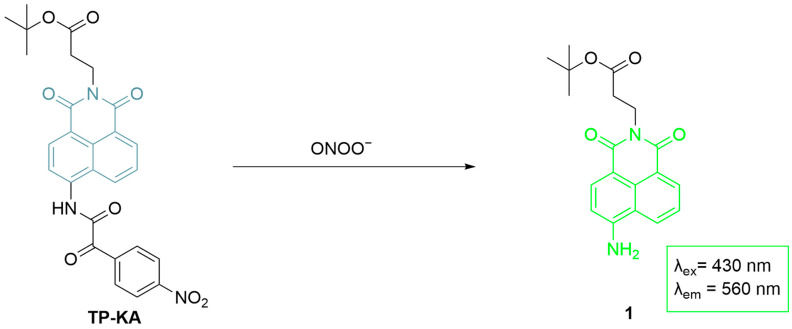
Schematic illustration of the sensing mechanism of **TP-KA** for ONOO^−^.

Li et al. developed **TP-KA**, a two-photon fluorescence probe for detecting drug-induced hepatotoxicity by monitoring ONOO^−^ levels (Figure 22) [81]. **TP-KA**, synthesized by attaching an α-ketoamide group to 1,8-naphthalimide, enables two-photon excitation and deep tissue imaging, with an additional tert-butyl ester group to improve cell membrane penetration. The probe achieved selective detection of ONOO^−^ with an LoD of 25 nM. In HepG2 cells, **TP-KA** achieved the detection of **APAP**-induced ONOO^−^ upregulation. In addition, **TP-KA** revealed elevated ONOO^−^ levels in **APAP** and tolcapone-induced liver injury, and a reduced fluorescence was observed after N-Acetyl-L-cysteine (**NAC**) treatment. **TP-KA** facilitates the real-time monitoring of hepatotoxicity, understanding toxicity mechanisms, and evaluating antidote efficacy.

**Figure 23:**
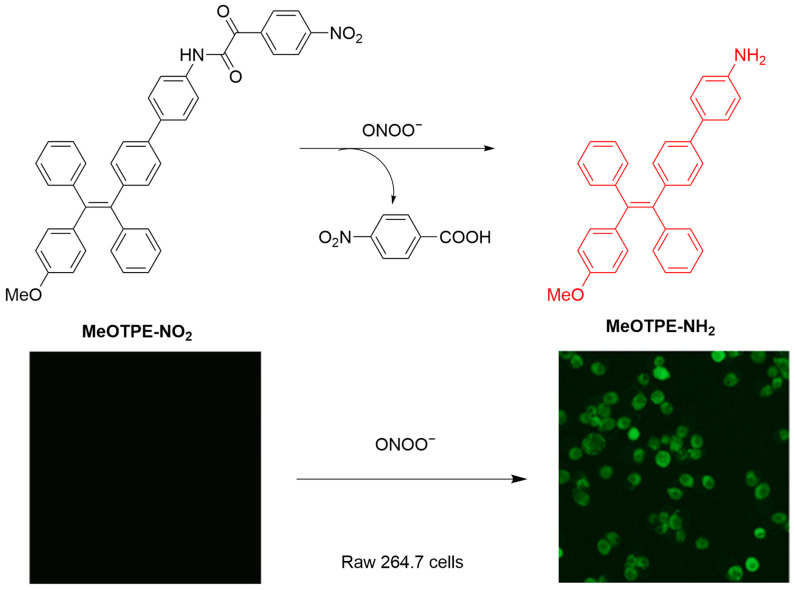
Schematic illustration of the sensing mechanism of **MeOTPE-NO_2_** for ONOO^−^. Reproduced with permission from [82].

Yan et al. developed a new fluorescent probe, **MeOTPE-NO_2_**, based on α-ketoamide, to selectively detect ONOO^−^ (Figure 23) [82]. The probe exhibits aggregation-induced emission (AIE) characteristics. Upon reaction with ONOO^−^, the probe is converted to **MeOTPE-NH_2_**, releasing strong fluorescence (λ_ex-max_/λ_em-max_ = 380 nm/505 nm). The fluorescence of the probe gradually enhanced in RAW 264.7 macrophages treated with increasing concentrations of ONOO^−^. In a mouse arthritis model, the probe achieved real-time in vivo imaging, revealing a significantly increased ONOO^−^ level at the initial stage of inflammation and a decrease in fluorescence at late stages.

**Figure 24:**
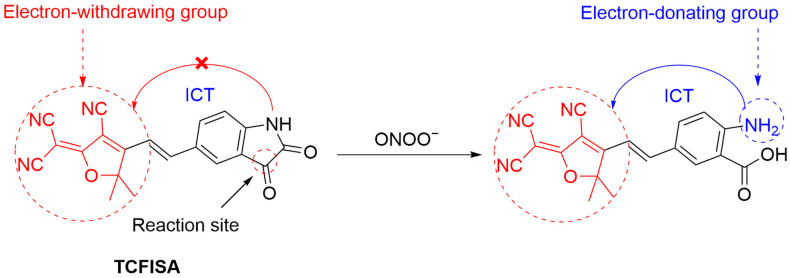
Schematic illustration of the sensing mechanism of the probe **TCFISA** for ONOO^−^.

Zhang et al. developed **TCFISA**, a fluorescent probe based on isatin for detecting intracellular ONOO^−^ at nanomolar levels (Figure 24) [83]. The probe, constructed by conjugating tricyanofuran (**TCF**) with isatin, undergoes oxidative decarbonylation in the presence of ONOO^−^, releasing a primary aniline with a λ_em-max_ at 606 nm. **TCFISA** has an LoD of 1.26 nM and a response time of <1 s. Its good cell membrane permeability allows for a sensitive and selective detection of ONOO^−^ in RAW 264.7 cells.

**Figure 25:**
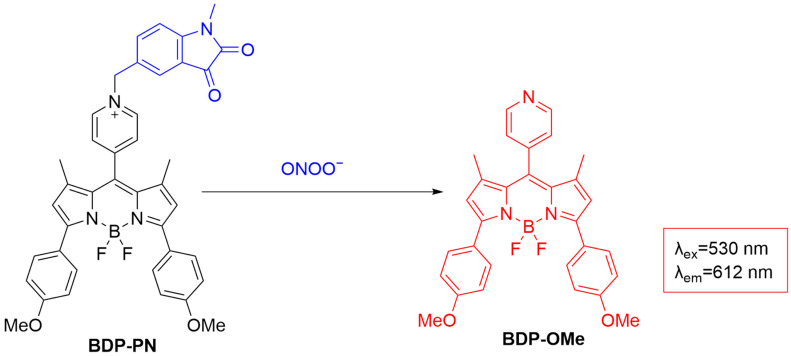
Schematic illustration of the sensing mechanism of the probe **BDP-PN** for ONOO^−^.

Qin et al. developed **BDP-PN**, a red fluorescent probe based on the coupling of an α-ketoamide to a boron-dipyrromethene (**BODIPY**) derivative, for monitoring drug-induced liver injury (DILI) (Figure 25) [77]. Being non-fluorescent due to the PeT effect, **BDP-PN** undergoes oxidation upon interaction with ONOO^−^, triggering a self-elimination reaction that enhances fluorescence (~60-fold) at λ_em-max_ of 612 nm. The fluorescence enhancement of the probe shows a strong linear relationship with ONOO^−^ concentration, with an LoD of 0.22 μM. In cellular studies, **BDP-PN** produced bright red fluorescence in various cell lines treated with **SIN-1** and **LPS**/**IFN-γ** and was used to differentiate between normal and cancer cells based on measuring endogenous ONOO^−^ levels. In a mouse model of **APAP**-induced liver injury, **BDP-PN** achieved the monitoring of the alternation in ONOO^−^ levels, aiding in the diagnosis of DILI.

**Figure 26:**
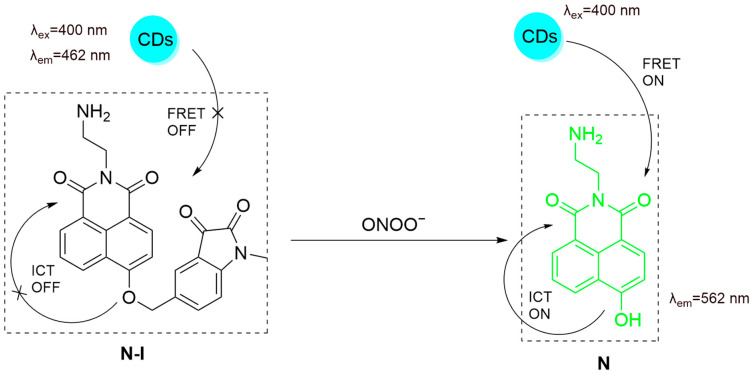
Schematic illustration of the sensing mechanism of **CD-N-I** for ONOO^−^.

Our group developed **CD-N-I**, a selective FRET nanoprobe based on the self-assembly between carbon dots (CDs) and a small-molecule fluorescent probe, naphthalimide–isatin (**N-I**), for ONOO^−^ detection (Figure 26) [84]. CDs act as energy donors, and naphthalimide as the acceptor, enhancing sensitivity and selectivity. ONOO^−^ reaction cleaves the isatin group, activating the FRET process to enable ratiometric sensing. The λ_em-max_ of the nanoprobe shifted from 462 nm to 562 nm after reaction with ONOO^−^ with a good linearity by plotting the fluorescence intensity ratio (I_562_/I_462_) as a function of ONOO^−^ concentration. In HepG2 cells, **CD-N-I** showed a significant increase in fluorescence ratio (I_562_/I_462_) in **APAP**-induced DILI. When cells were treated with **NAC**, minimal ratio increase was seen. **CD-N-I** could be used for monitoring DILI, exploiting the simple supramolecular association between small-molecule probes and photoluminescent carbon materials. Modifying the carbon materials with targeting agents such as carbohydrates could further improve liver targeting [85,86,87,88,89].

**Figure 27:**
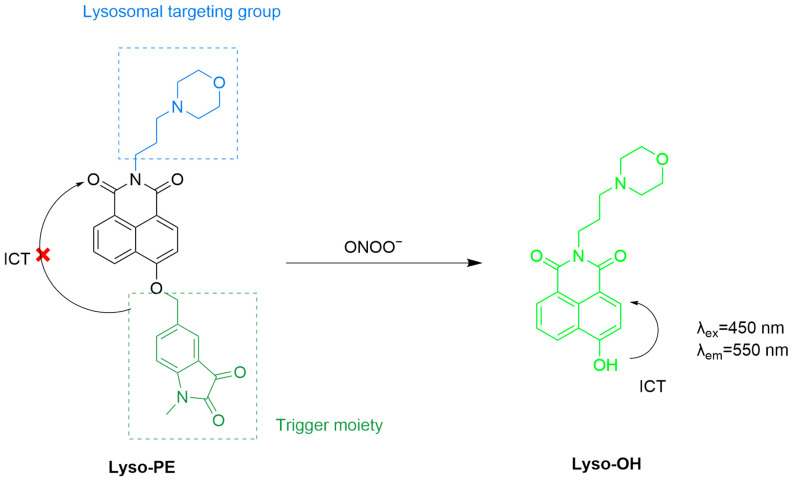
Schematic illustration of the sensing mechanism of **Lyso-PE** for ONOO^−^.

Jiang et al. developed a lysosomal-targeting fluorescent probe, **Lyso-PE**, for in vivo ONOO^−^ detection (Figure 27) [90]. The probe consists of 4-hydroxy-1,8-naphthalenediimide as fluorophore, a methylindigo moiety as the sensing unit, and a morpholine ring to target lysosomes. Upon reacting with ONOO^−^, the methylindigo group is cleaved, thus recovering the fluorescence of the system by setting free its ICT process. In 4T1 cells treated with increasing concentrations of ONOO^−^, the fluorescence intensity of the probe was gradually enhanced. Intratumoral injection of the probe in a 4T1 tumor-bearing mouse model led to a significantly higher fluorescence in the tumor area than in normal tissues being seen, suggesting the potential of the probe for use in fluorescence-guided surgery of tumors.

### 3.4. Fluorescent Probes Based on Thioethers

**Figure 28:**
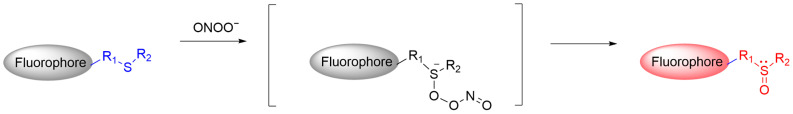
Schematic illustration of the reaction mechanism between thioether-based fluorescent probes and ONOO^−^.

Thioethers are electron-rich with low redox potentials, which can react with ONOO^−^, effectively leading to structural changes in sulfide, thus enhancing the fluorescence of conjugated dyes through ICT or PeT (Figure 28) [91].

**Figure 29:**
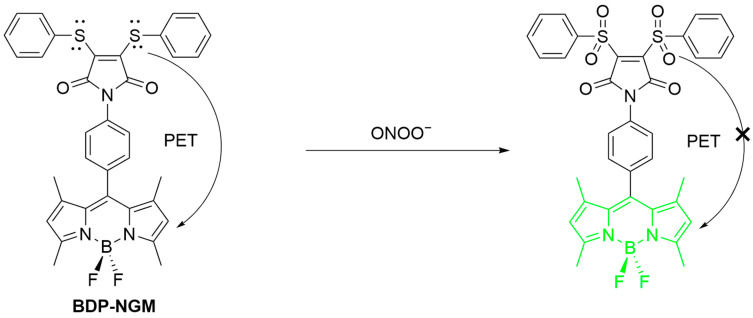
Schematic illustration of the sensing mechanism of **BDP-NGM** for ONOO^−^.

Yudhistira et al. developed **BDP-NGM**, a fluorescent probe for selective ONOO^−^ detection (Figure 29) [92]. The probe’s dithiomaleimide moiety reacts with ONOO^−^, oxidizing sulfur to sulfone to significantly enhance fluorescence. With excitation at 502 nm, a λ_em-max_ of 512 nm was detected, with an increase in quantum yield from 0.0052 to 0.42 upon reaction with ONOO^−^. The probe shows strong green fluorescence with ONOO^−^ in cells, but not with other ROS like H_2_O_2_ and NaOCl, offering a sensitive and selective tool for real-time ONOO^−^ monitoring in cells.

### 3.5. Fluorescent Probes Based on Conjugated Double Bonds (C=C/C=N)

**Figure 30:**
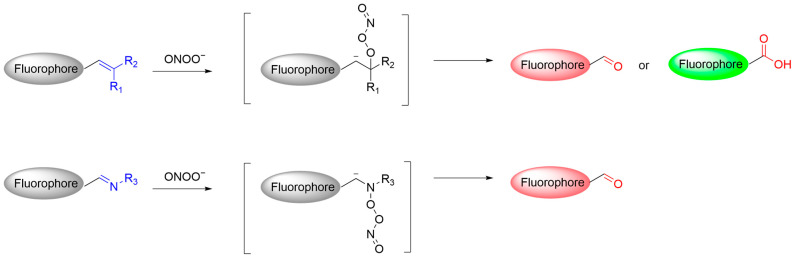
Schematic illustration of the reaction mechanism between fluorescent probes based on conjugated double bonds and ONOO^−^.

ONOO^−^ is a potent oxidative capable of effectively cleaving carbon–carbon (C=C) and carbon–nitrogen (C=N) double bonds through nucleophilic attack. Therefore, fluorescent probes based on these unsaturated bonds have been developed (Figure 30) [93].

#### 3.5.1. Fluorescent Probes Based on C=C Bonds

**Figure 31:**
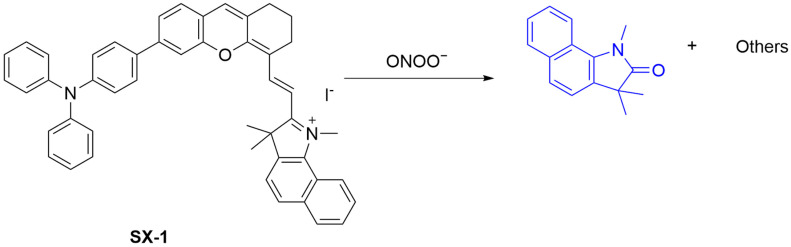
Schematic illustration of the sensing mechanism of the probe **SX-1** for ONOO^−^.

Cyclophosphamide, a chemotherapeutic drug, increases intracellular oxidative stress as marked by an elevated level of ONOO^−^. Li et al. developed **SX-1**, a fluorescent probe for detecting ONOO^−^ in lipid droplets (LDs) during cyclophosphamide treatment (Figure 31) [94]. **SX-1**, featuring a triphenylamine-benzoindocyanine moiety, targets LDs and emits blue fluorescence at a λ_em-max_ of 456 nm when its C=C bond is cleaved by ONOO^−^. The probe has an LoD of 326 nM and shows high selectivity and sensitivity. In HeLa cells simulated by **LPS** and **IFN-γ**, **SX-1** achieved the detection of ONOO^−^ with fluorescence enhancement; in contrast, the fluorescence enhancement was compromised by the addition of uric acid, suggesting its ability to monitor the dynamic change in intracellular ONOO^−^ levels. This study offers a chemical tool for studying the mechanism of action of cyclophosphamide.

**Figure 32:**
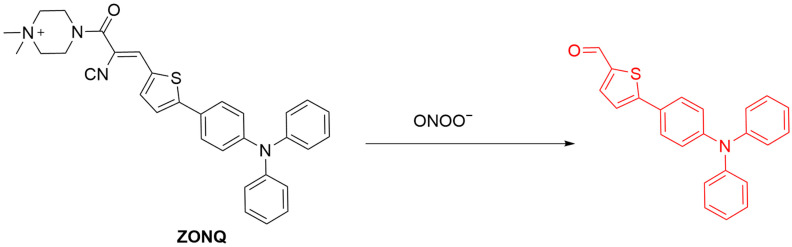
Schematic illustration of the sensing mechanism of the probe **ZONQ** for ONOO^−^.

ONOO^−^ has been reported to be upregulated in glioblastoma [95]. Zhang et al. developed **ZONQ**, which is capable of detecting ONOO^−^, aiding in the real-time diagnosis of glioblastoma (Figure 32) [96]. **ZONQ** reacts with ONOO^−^ by oxidatively cleaving its double bond to release fluorophore **ZOQ**, resulting in a substantial fluorescence enhancement at a λ_em-max_ of 548 nm. The probe is selective for ONOO^−^ over other ROS tested, with an LoD of 0.9 µM. The cell-imaging capacity of **ZONQ** in the presence of ONOO^−^ was demonstrated in U87MG (human malignant glioblastoma) cells. In addition, using an orthotopic xenograft model of nude mice, **ZONQ** injected via the tail vein showed an enhanced fluorescence at the tumor site in the brain, indicating its ability to monitor ONOO^−^ accumulation in tumor tissue.

**Figure 33:**
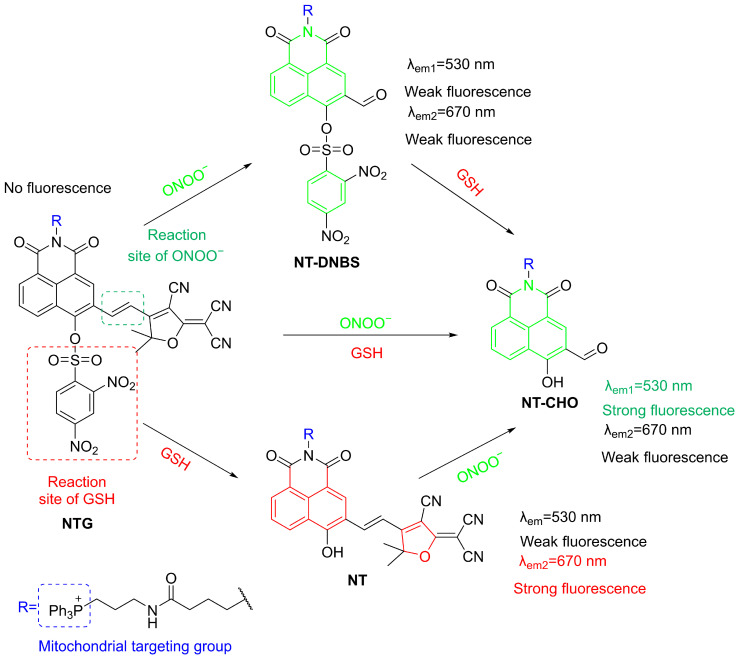
Schematic illustration of the sensing mechanism of the probe **NTG** for GSH and ONOO^−^.

A multifunctional probe, **NTG**, was developed by Luo et al., enabling simultaneous detection of ONOO^−^ and glutathione (GSH) in mitochondria (Figure 33) [97]. **NTG** consists of a 1,8-naphthalimide fluorophore, a GSH-responsive 2,4-dinitrobenzenesulfonyl group, and a tri-phenylphosphine mitochondrial-targeting group. GSH induces a nucleophilic aromatic substitution, resulting in a red fluorescence at a λ_em-max_ of 670 nm being seen. Subsequently, oxidative cleavage of the probe caused by ONOO^−^ further leads to a blue-shift of fluorescence maximum to 530 nm. In MCF-10A (human mammary epithelial) cells, **NTG** showed intense red fluorescence with GSH addition, and a subsequent upregulation of ONOO^−^ in cells activated the blue-shifted fluorescence. The dual-channel fluorescence of **NTG** achieving the effective tracking of mitochondrial GSH and ONOO^−^ dynamics in live cells and zebrafish made possible the classification of different stages of breast cell progression, thus potentially aiding in early breast cancer diagnosis.

**Figure 34:**
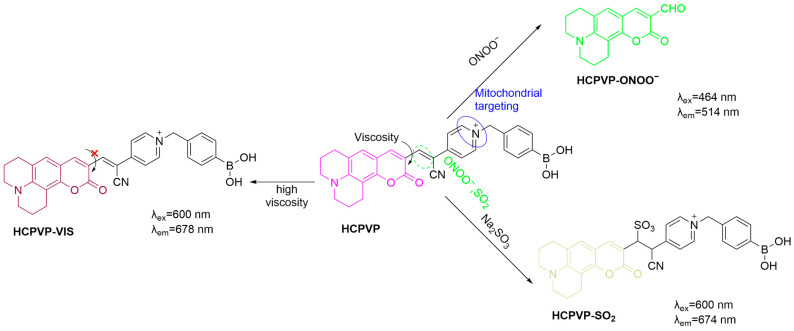
Schematic illustration of the sensing mechanism of **HCPVP** for ONOO^−^, SO_2_, and viscosity.

Li et al. further developed a three-channel fluorescent probe, **HCPVP**, for mitochondrial detection of ONOO^−^, SO_2_, and viscosity (Figure 34) [98]. **HCPVP** consists of a coumarin fluorophore and a C=C bond reactive to ONOO^−^. The presence of ONOO^−^ causes oxidative cleavage of the probe, leading to the enhancement of a yellow fluorescence at a λ_em-max_ of 514 nm with an LoD of 85 nM. On the other hand, cell viscosity can cause the fluorescence of the “D-π-A”-type probe to red-shift to a λ_em-max_ of 678 nm, while SO_2_ induces fluorescence quenching of the probe through a Michael addition reaction. **HCPVP** was shown to be effective in monitoring these factors in cells and zebrafish. In a collagen-induced arthritis mouse model, **HCPVP** was used to image early RA joint changes, aiding in understanding RA pathogenesis and evaluating anti-inflammatory treatments.

**Figure 35:**
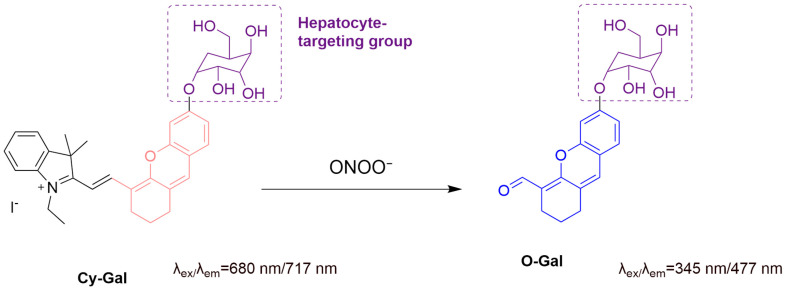
Schematic illustration of the sensing mechanism of the probe **Cy-Gal** for ONOO^−^.

Li et al. developed **Cy-Gal**, a hepatocyte-targeting ratiometric fluorescent probe for detecting ONOO^−^ in liver injury (Figure 35) [99]. The probe, containing a hemicyanine fluorophore modified by galactose, targets the asialoglycoprotein receptor (ASGPr) expressed by hepatocytes and shows red fluorescence at a λ_em-max_ of 717 nm. The presence of ONOO^−^ oxidatively cleaves the C=C bond, blue-shifting the fluorescence to a λ_em-max_ of 477 nm, thus enabling sensing in a ratiometric manner. **Cy-Gal** has an LoD of 0.18 μM and can effectively monitor ONOO^−^ in HepG2 cells treated with **SIN-1** or **LPS**. The probe also achieved liver-specific ONOO^−^ imaging in a mouse model of liver injury induced by carbon tetrachloride or rifampicin.

**Figure 36:**
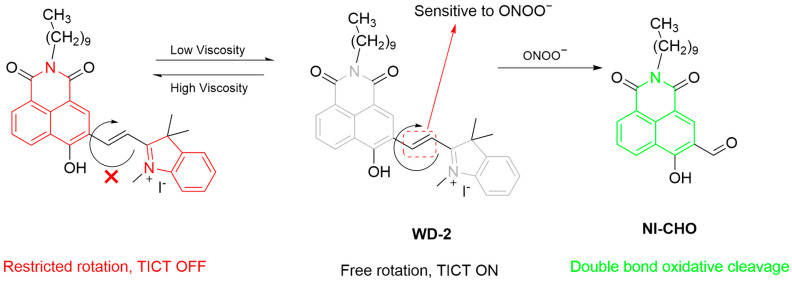
Schematic illustration of the sensing mechanism of the probe **WD-2** for ONOO^−^.

The interaction between lipid droplets (LDs) and mitochondria is crucial for cellular energy metabolism and homeostasis. To expand the application of ONOO^−^ fluorescent probes incorporating C=C bonds, Wei et al. developed a multifunctional fluorescent probe, **WD-2**, that is “double-responsive” to viscosity and ONOO^−^ and “dual-targeting” to LDs and mitochondria for detecting ONOO^−^ level changes in mitochondria during cell pyroptosis (Figure 36) [100]. Upon reacting with ONOO^−^, the double bond in **WD-2** undergoes oxidative cleavage, forming **NI-CHO** with a significantly enhanced green fluorescence. On the other hand, the fluorescence of the probe at a λ_em-max_ of 620 nm significantly enhanced as viscosity increased. **WD-2** was shown to effectively target mitochondria and LDs in HepG2 cells, achieving the detection of ONOO^−^ level changes in cisplatin- and adriamycin-induced pyroptosis models. In a zebrafish model, **WD-2** similarly visualized viscosity and ONOO^−^ changes with dual fluorescence.

**Figure 37:**
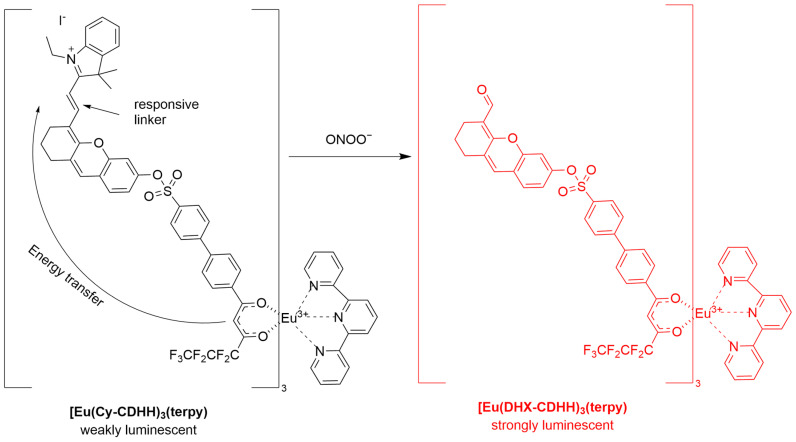
Schematic illustration of the sensing mechanism of the probe **Eu(Cy-CDHH)_3_(terpy)** for ONOO^−^.

Zou et al. developed a novel activatable β-diketonate europium(III) complex-based probe, **Eu(Cy-CDHH)_3_(terpy)**, for time-gated luminescence detection of ONOO^−^ (Figure 37) [101]. Upon reaction with ONOO^−^, the unsaturated double bond of the phlorocyanine dye in **Eu(Cy-CDHH)_3_(terpy)** is cleaved, restoring the strong, long-lived luminescence of the β-diketonate-Eu^3+^ complex, with a significantly enhanced emission at 606 nm (∼600-fold enhancement). In HepG2 cells, **Eu(Cy-CDHH)_3_(terpy)** effectively detected both exogenous ONOO^−^ induced by **SIN-1** and endogenously produced ONOO^−^ as simulated by **LPS** and **IFN-γ**. The probe demonstrated sensitivity and specificity in a living cell model of **APAP**-induced liver injury and a BALB/c mouse model of liver injury.

#### 3.5.2. Fluorescent Probes Based on C=N Bonds

**Figure 38:**
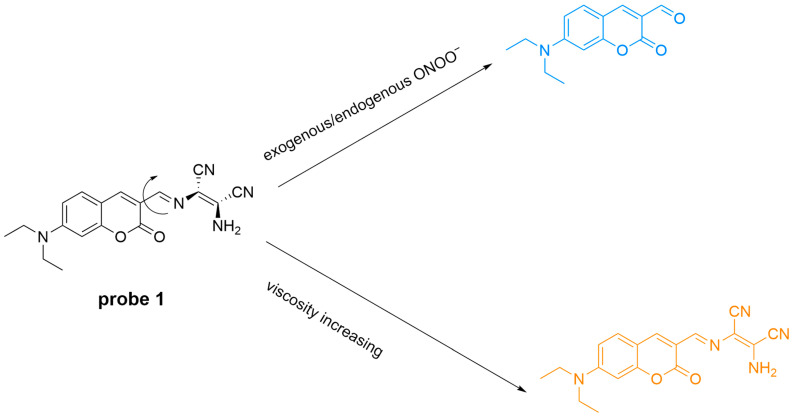
Schematic illustration of the sensing mechanism of **Probe 1** for ONOO^−^ and viscosity.

Wang et al. developed a dual-responsive fluorescent probe (**Probe 1**) for imaging both intracellular viscosity and ONOO^−^ (Figure 38) [102]. Under low viscosity, the probe shows weak fluorescence but emits at a λ_em-max_ of 571 nm as viscosity increases. ONOO^−^ cleaves the C=N bond, causing a blue-shift of λ_em-max_ to 504 nm. The probes with high sensitivity and selectivity successfully detected ONOO^−^ and viscosity changes in HeLa cells and monitored ONOO^−^ variations in zebrafish induced by **LPS** and **IFN-γ**.

**Figure 39:**
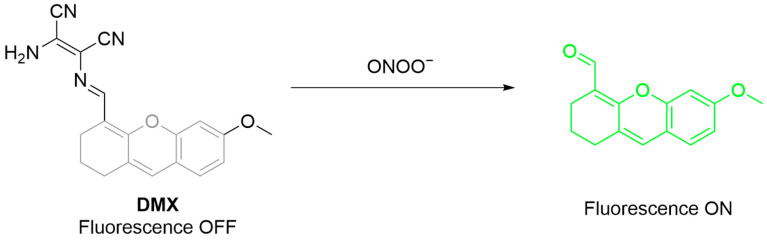
Schematic illustration of the sensing mechanism of the probe **DMX** for ONOO^−^.

Liang et al. developed **DMX** for detecting ONOO^−^ in cells and zebrafish models (Figure 39) [103]. The probe, consisting of a xanthene core with a C=N bond as the recognition site, shows minimal baseline fluorescence. The presence of ONOO^−^ causes oxidative cleavage of the C=N bond, enhancing the fluorescence of the probe at a λ_em-max_ of 510 nm. The probe is sensitive (LoD 37 nM) and selective over other ROS. It also effectively monitored **SIN-1**-simulated ONOO^−^ in HeLa cells and zebrafish.

### 3.6. Fluorescent Probes Based on Trifluoromethyl Ketone

**Figure 40:**
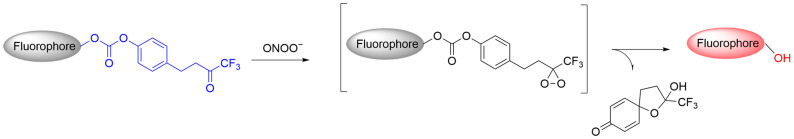
Schematic illustration of the sensing mechanism of trifluoromethyl-ketone-based probes for ONOO^−^.

ONOO^−^ can react with activated ketone to form a diketone, a process analogous to the reaction between ketones and peroxymonosulfate (Figure 40) [104]. Upon reaction with ONOO^−^, a fluorogenic probe that contains a trifluoromethyl ketone group is first converted to a dioxane intermediate, which then undergoes selective oxidation, producing a diketone product and the fluorescent species [105].

**Figure 41:**
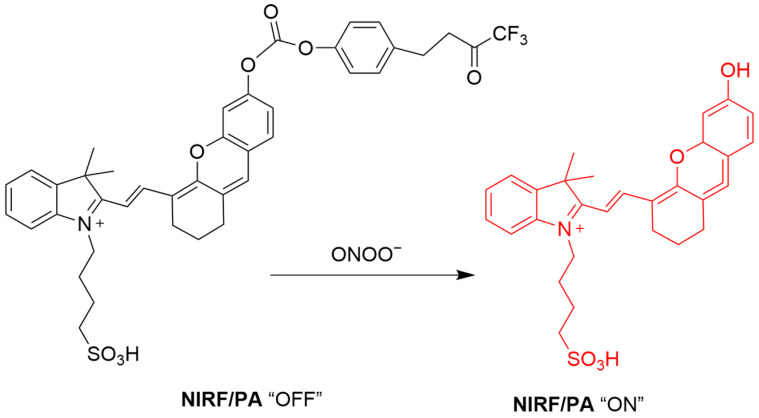
Schematic illustration of the sensing mechanism of the probe **CySO3CF3** for ONOO^−^.

Zhang et al. developed **CySO3CF3** with NIR fluorescence and photoacoustic properties for in vivo imaging of ONOO^−^ in solid tumors (Figure 41) [106]. The probe, consisting of the ONOO^−^-responsive trifluoromethyl ketone group and a hemicyanine dye, is initially non-fluorescent. ONOO^−^ exposure triggers oxidative reactions, converting **CySO3CF3** to **CySO3OH** with fluorescence enhancement at a λ_em-max_ of 712 nm and generation of photoacoustic signal. The probe was used to obtain fluorescence-based imaging of ONOO^−^ in RAW 264.7 cells and dual-modality (fluorescence and photoacoustic) imaging of ONOO^−^ in a breast-cancer-cell-line-derived xenograft murine model.

**Figure 42:**
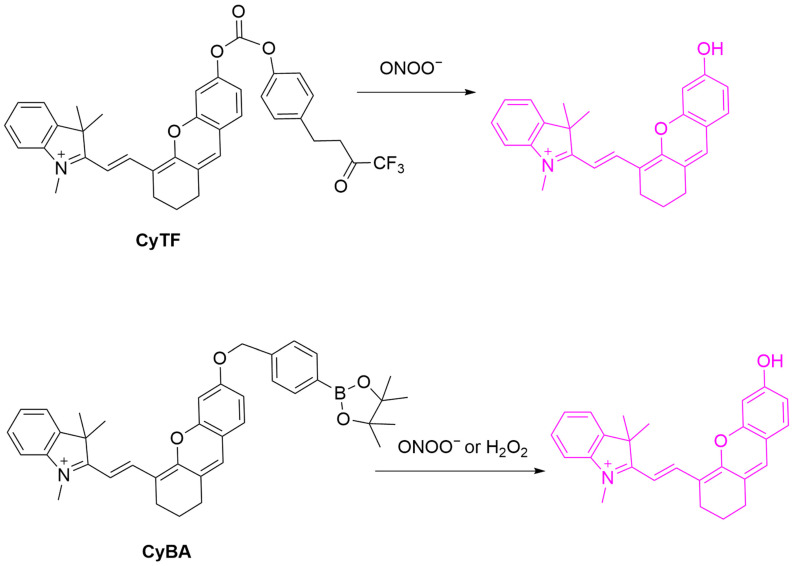
Schematic illustration of the sensing mechanisms of **CyTF** and **CyBA** for ONOO^−^.

Cheng et al. developed two ONOO^−^-responsive probes, **CyTF** and **CyBA**, for keloid imaging (Figure 42) [107]. **CyTF**, with a trifluoromethyl ketone group, and **CyBA**, with a borate group, are both non-fluorescent initially. ONOO^−^ exposure sets free the fluorescence of both **CyTF** and **CyBA** probes with a fluorescence enhancement of up to 15-fold at a λ_em-max_ of 717 nm. The selectivity of **CyTF** was shown to be better than that of **CyBA** over H_2_O_2_. In cellular experiments, both probes showed fluorescence enhancement in TGF-β1-stimulated normal dermal fibroblasts (NDFs) and keloid fibroblasts (KFs). The fluorescence of **CyTF** was reduced by the TβR1 inhibitor RepSox, suggesting its usefulness for drug screening. In a mouse model established by a KF xenograft tumor, **CyTF** exhibited intense fluorescence at keloid sites, suggesting ONOO^−^ upregulation during keloid pathogenesis.

**Figure 43:**
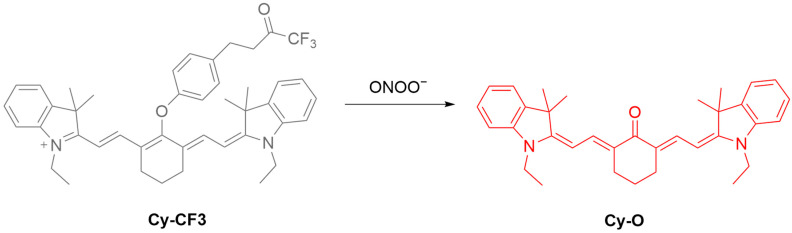
Schematic illustration of the sensing mechanism of the probe **Cy-CF3** for ONOO^−^.

Huang et al. developed **Cy-CF3**, an ONOO^−^-responsive NIR fluorescent probe, to study the relationship between hypoxia and ONOO^−^ (Figure 43) [108]. The probe features a heptamethine cyanine dye, whose fluorescence is quenched by a trifluoroketone group responsive to ONOO^−^. ONOO^−^ exposure causes oxidative cleavage, leading to fluorescence enhancement of the probe at a λ_em-max_ of 630 nm with an LoD of 9.2 nM. In cellular experiments, the fluorescence enhancement of **Cy-CF3** in **SIN-1**-simulated LO2 (human liver) cells was suppressed by an ONOO^−^ scavenger, minocycline. The fluorescence of the probe was shown to correlate with the hypoxic levels in LO2 cells and zebrafish models. In a mouse model of acute hepatic ischemia, **Cy-CF3** achieved monitoring of ONOO^−^ changes in real time.

### 3.7. Fluorescent Probes Based on Aromatic Compounds (Hydroquinone, Benzopyrylium/Benzopyran)

#### 3.7.1. Fluorescent Probes Based on Hydroquinone

**Figure 44:**
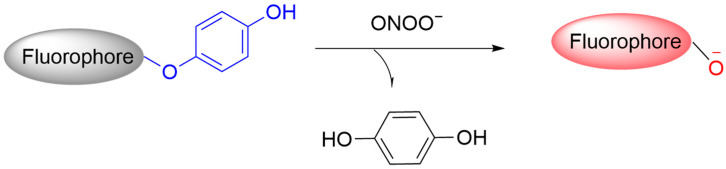
Schematic illustration of the sensing mechanism of hydroquinone-based probes for ONOO^−^.

ONOO^−^ can directly oxidize electron-rich functional groups through one- or two-electron transfer processes (Figure 44) [109]. Phenol and aniline derivatives, which are highly electron-rich, can be readily oxidized by ONOO^−^. The integration of these groups into fluorescent molecules facilitates the development of new types of ONOO^−^-responsive probes. In the presence of ONOO^−^, these fluorogenic probes can undergo oxidative N-dearomatization to restore fluorescence [110].

**Figure 45:**
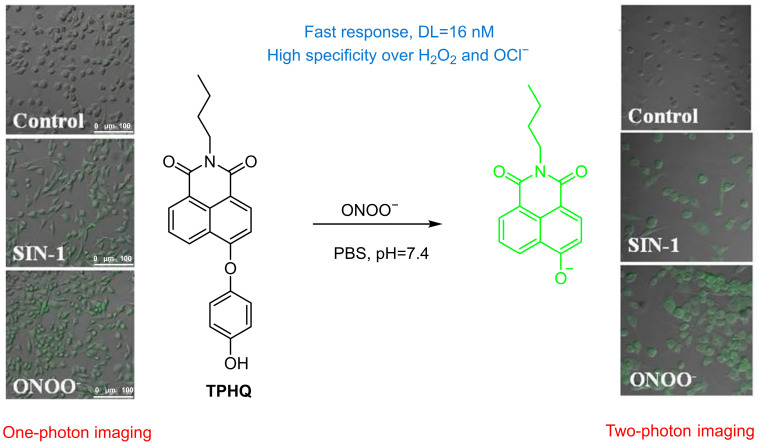
Schematic illustration of the sensing mechanism of the probe **TPHQ** for ONOO^−^. Reproduced with permission from [111].

Zhu et al. developed **TPHQ**, a two-photon, NIR fluorescent probe based on 4-hydroxynaphthalimide for ONOO^−^ detection in living cells (Figure 45) [111]. **TPHQ** features 4-hydroxynaphthalimide as the fluorophore and hydroquinone as the ONOO^−^ recognition unit. Reaction with ONOO^−^ releases the fluorophore with a significantly enhanced fluorescence at a λ_em-max_ of 550 nm. The probe has a high sensitivity (LoD = 16 nM) and selectivity over H_2_O_2_ and OCl^−^. In RAW 264.7 macrophages, **TPHQ** showed substantially enhanced green fluorescence in cells treated with **SIN-1**.

**Figure 46:**
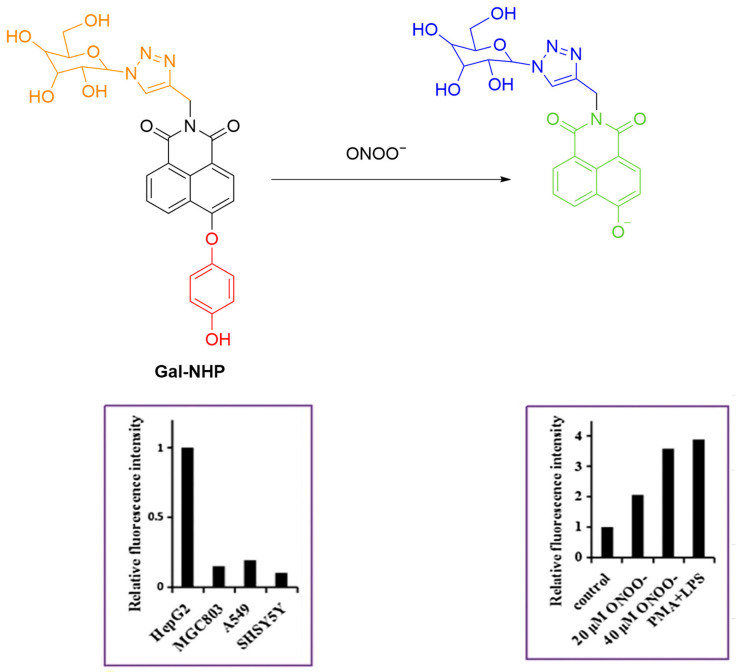
Schematic illustration of the sensing mechanism of **Gal-NHP** for ONOO^−^. Reproduced with permission from [112].

Liu et al. developed **Gal-NHP**, a galactose-modified, ASPGr-targeting fluorescent probe for monitoring ONOO^−^ in HepG2 cells (Figure 46) [112]. The introduction of the galactosyl group improves water solubility and facilitates targeted accumulation of the probe in HepG2 cells. Upon reaction with ONOO^−^, **Gal-NHP** undergoes a structural transformation by separating into a hydroquinone and the fluorescent hydroxynaphthalimide with a strong fluorescence at a λ_em-max_ of 555 nm. Cellular experiments confirmed that **Gal-NHP** selectively imaged ONOO^−^ in HepG2 cells via ASGPR-mediated endocytosis.

**Figure 47:**
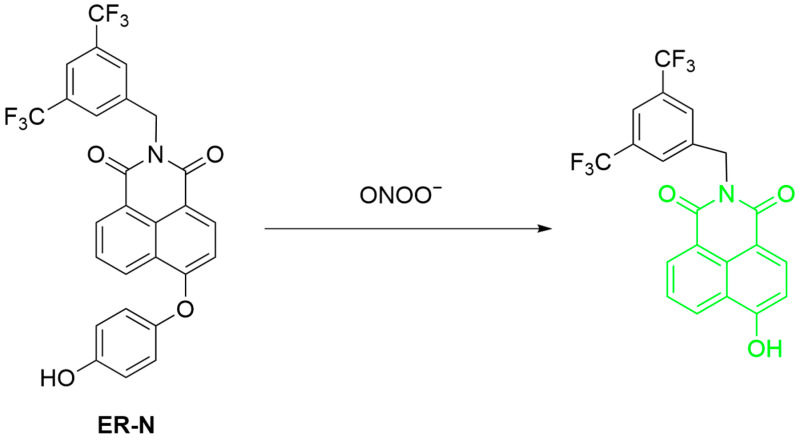
Schematic illustration of the sensing mechanism of the probe **ER-N** for ONOO^−^.

Based on the above research basis of their research group, Liu et al. further developed **ER-N** for ONOO^−^ detection in the endoplasmic reticulum (ER) during DILI (Figure 47) [113]. **ER-N** consists of naphthylamide as fluorophore, hydroquinone to react with ONOO^−^, and bis(trifluoromethyl)benzene to target ER. Hydroquinone release of the probe after reaction with ONOO^−^ led to a fluorescence restoration at a λ_em-max_ of 550 nm. The probe is selective over other ROS with a 43 nM LoD. It achieved imaging of ONOO^−^ in HeLa cells, zebrafish, and a mouse model of **APAP**-induced liver injury.

**Figure 48:**
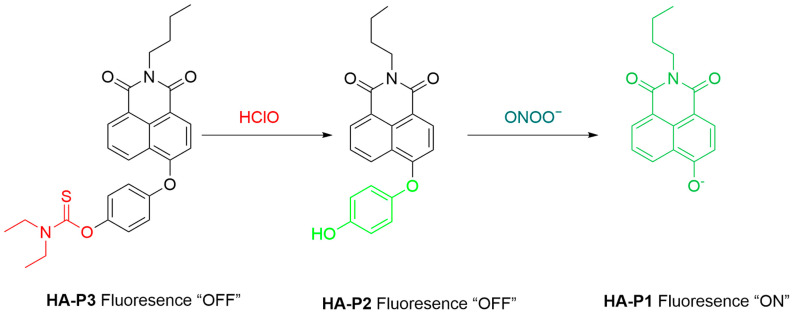
Schematic illustration of the sensing mechanism of the probe **HA-P3** for ONOO^−^.

Recently, Liu et al. developed a sequence-activatable dual-locked fluorescent probe, **HA-P3**, to improve DILI diagnosis by the simultaneous detection of two DILI-associated biomarkers, hypochlorous acid (HOCl) and ONOO^−^ (Figure 48) [114]. **HA-P3** first reacts with HOCl via the diethyl thiocarbamate moiety to form **HA-P2** with a released hydroquinone group to sequentially react with ONOO^−^. In RAW264.7 cells pretreated with SIN-1 and subsequently exposed to HOCl and **HA-P3**, significant fluorescence signals were observed, indicating the effectiveness of **HA-P3** to simultaneously detect HOCl and ONOO^−^ in live cells. In APAP-induced liver injury mouse and zebrafish models, **HA-P3** also achieved the imaging of HOCl and ONOO^−^ level changes during DILI. This sequential sensing strategy might overcome false-positive signals derived from fluorescent probes responsive to just a single analyte.

**Figure 49:**
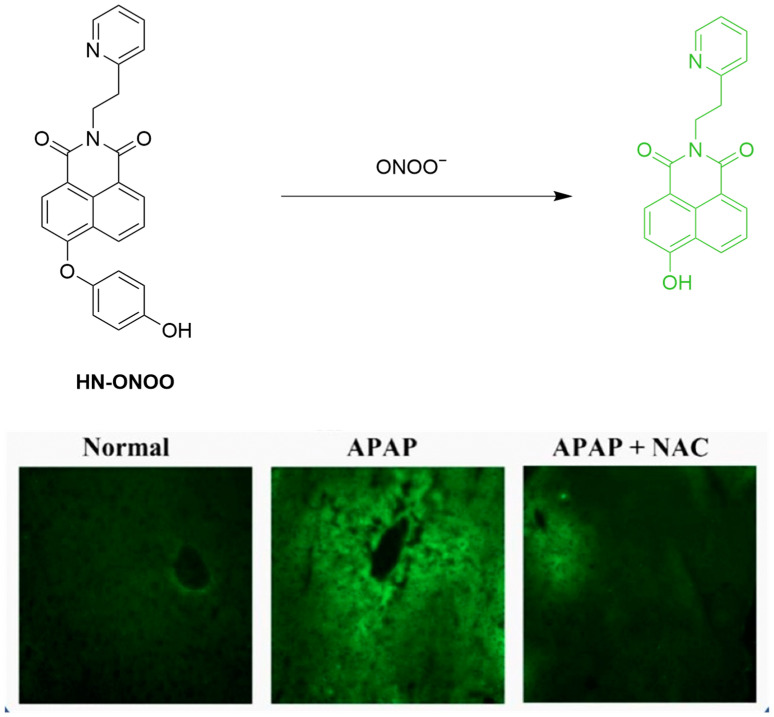
Schematic illustration of the sensing mechanism of **HN-ONOO** for ONOO^−^. Reproduced with permission from [115].

Wei et al. developed **HN-ONOO** similarly based on naphthylimide for monitoring ONOO^−^ during liver injury (Figure 49) [115]. Initially non-fluorescent, **HN-ONOO** reacts with ONOO^−^ to release the hydroquinone group. This gives rise to fluorescence restoration of naphthylimide at a λ_em-max_ of 542 nm with a broad linear detection range (0–125 μM) and rapid response time (within 1 min). Cellular experiments in HepG2 and LO2 cells pretreated with a variety of agents to upregulate intracellular ONOO^−^ demonstrated the capacity of the probe for monitoring the RNS in live cells. **HN-ONOO** also achieved ONOO^−^ imaging in **APAP**-treated liver tissues, suggesting its potential for diagnosis of liver injury.

#### 3.7.2. Fluorescent Probes Based on Benzopyrylium/Benzopyran

**Figure 50:**
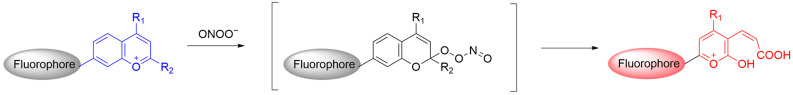
Schematic illustration of the sensing mechanism of benzopyrylium/benzopyran-based probes for ONOO^−^.

Benzopyrylium or benzopyran is electron-deficient, making it prone to sequential oxidation and hydrolytic reactions with ONOO^−^. This process can lead to the release of fluorescent groups (Figure 50) [116].

**Figure 51:**
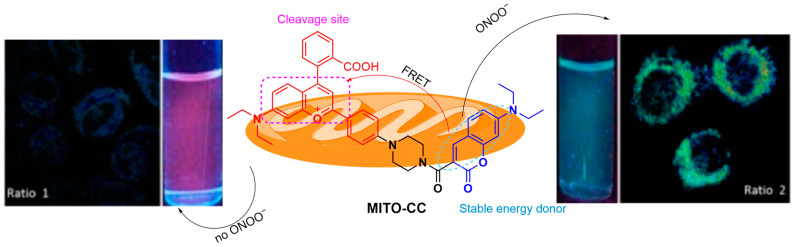
Schematic illustration of the detection mechanism of the probe **MITO-CC** for ONOO^−^. Reproduced with permission from [116].

Cheng et al. developed **MITO-CC**, a FRET-based, mitochondrial-targeting ratiometric fluorescent probe for detecting ONOO^−^ (Figure 51) [116]. **MITO-CC** consists of coumarin as the FRET donor and benzopyrylium as the acceptor, exhibiting strong emission at a λ_em-max_ of 651 nm without ONOO^−^. In the presence of ONOO^−^, the coumarin fluorescence at a λ_em-max_ of 473 nm is enhanced, with benzopyrylium fluorescence at a λ_em-max_ of 651 nm concurrently decreasing. The LoD of the probe was measured to be 11.30 nM. **MITO-CC** was shown to be localized in the mitochondria of HepG2 and HeLa cells with the locally generated ONOO^−^ as simulated by **LPS** and **IFN-γ** being detectable by coumarin fluorescence. The probe was also successfully used for ONOO^−^ imaging in a mouse inflammation model induced by subcutaneous injection of **LPS**.

**Figure 52:**
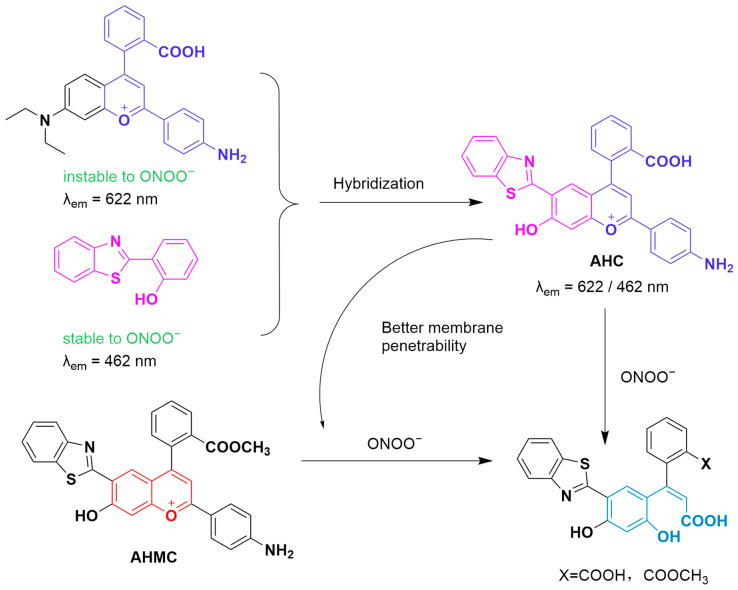
Schematic illustration of the structures of probes **AHC** and **AHMC** and the sensing mechanism for ONOO^−^.

Gong et al. developed two ratiometric fluorescent probes, **AHC** and **AHMC**, for detecting ONOO^−^ using a “molecular hybridization” strategy (Figure 52) [117]. **AHC** incorporates an ONOO^−^-reactive dye (NH_2_-benzopyrylium, λ_em-max_ of 626 nm) with an ONOO^−^-stable benzothiazole derivative (λ_em-max_ of 462 nm). Then, the ester form (**AHMC**) of the dye conjugate was synthesized to enhance cell membrane penetrability. The red fluorescence of the probe blue-shifts upon ONOO^−^ reaction, enabling the ratiometric detection of the RNS. In cellular experiments, **AHMC** successfully imaged intracellularly produced ONOO^−^ in **LPS**/**IFN-γ**-pretreated RAW264.7 cells, with a 3.5-fold increase in the blue/red fluorescence ratio. The probe also achieved ONOO^−^ monitoring in a cell model of LO2 mimicking acute liver injury pretreated with **APAP**, **LPS**, and isoniazid.

**Figure 53:**
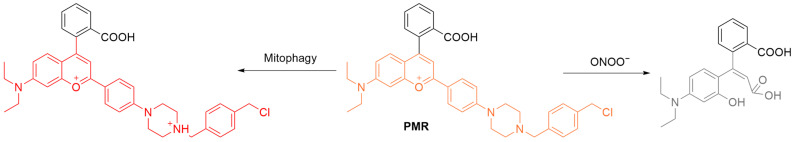
Schematic illustration of the sensing mechanism of the probe **PMR** for ONOO^−^ and mitophagy.

Li et al. developed **PMR**, a piperazine-based, mitochondrial-targeting fluorescent probe for simultaneous detection of ONOO^−^ and monitoring of mitochondrial autophagy (Figure 53) [118]. **PMR** shows enhanced fluorescence at a λ_em-max_ of 640 nm under mitochondrial acidification, and the fluorescence decreases upon generation of ONOO^−^ due to oxidative cleavage of the benzopyrylium moiety by the RNS. In cellular models, **PMR** effectively imaged ONOO^−^ and tracked mitochondrial autophagy induced by rapamycin or starvation.

**Figure 54:**
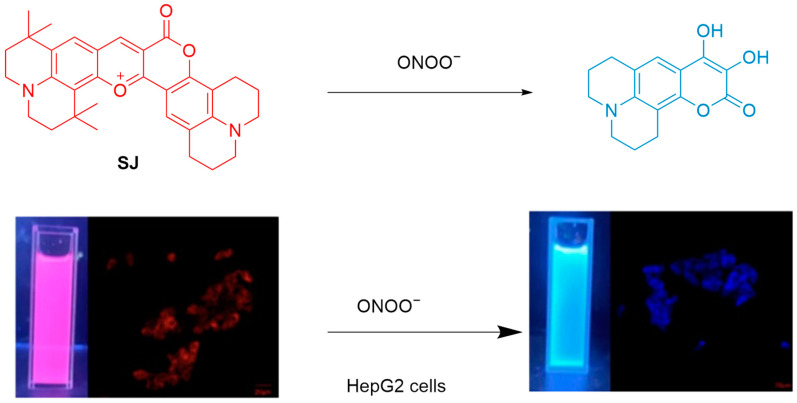
Schematic illustration of the sensing mechanism of the probe **SJ** for ONOO^−^. Reproduced with permission from [119].

Sun et al. developed **SJ**, a ratiometric fluorescent probe based on benzopyran, for detecting ONOO^−^ (Figure 54) [119]. **SJ**, synthesized by fusing a coumarin derivative with tetramethyljulolidine, uses benzopyran as both a fluorophore and recognition site. Reaction with ONOO^−^ causes benzopyran cleavage, thus enhancing the fluorescence of the probe at a λ_em-max_ of 451 with a concurrently decrease in fluorescence intensity at a λ_em-max_ of 688 nm. **SJ** features a large Stokes shift of 237 nm, a fast response time (≤10 s), a broad linear range (0–8.5 μM), selectivity over other ROS, and an LoD of 21.3 nM. It also achieved ONOO^−^ imaging in HepG2 cells.

## 4. Discussion

Boronate-based probes typically are structures where one or more boron atoms are bonded to hydroxyl groups. Their advantage lies in the ability of boronates to form stable complexes with ONOO^−^ through hydrogen bonds, thereby enhancing selectivity and sensitivity. Additionally, the reaction rate of boronic acids or boronic esters with ONOO^−^ is millions of times faster than that with H_2_O_2_, making them a good choice for designing ONOO^−^ probes. Aryl boronic esters are more often chosen as the responsive group, as the reaction rate of aliphatic boronates with ONOO^−^ is significantly lower than that of aromatic analogs [46]. Aryl-boronic-ester-based probes are also more stable and can therefore better maintain their structural integrity under harsh conditions. However, the selectivity of aryl boronic acids/esters for ONOO^−^ over other ROS/RNS is not optimal, necessitating further modification for improved selectivity.

Amine-based probes such as those based on acylhydrazines and ketoamides are also commonly developed for ONOO^−^ sensing. Hydrazine, due to its strong nucleophilicity, can rapidly react with ONOO^−^ to timely monitor its dynamic production under various biological conditions [120]. The selectivity of the reaction between hydrazine and ONOO^−^ over HClO can be controlled by the reactivity of the hydrazine group through its conjugation to different fluorophores. The commonly used fluorophores to which hydrazine is introduced include rhodamine and coumarin derivatives. However, the majority of fluorescent probes developed are excited in the visible light range, making the development of long-wavelength and NIR probes with improved penetration depth for in vivo applications necessary. Ketoamide probes, containing ketone and amide groups, are generally incorporated into fluorescent groups such as hemicyanine and 1,8-naphthylimide [81,121,122]. Both hydrazine and ketoamide probes are promising for ONOO^−^ detection in terms of selectivity and sensitivity.

Double-bond-based probes typically contain one or more unsaturated carbon chains. The highly oxidative ONOO^−^ can react with double-bond-containing compounds rapidly [123]. Double-bonded compounds can also be used for the simultaneous detection of ONOO^−^ and another stimulus such as viscosity, making the resulting sensing applications more accurate and versatile. However, to improve the stability and selectivity of double-bond-based probes, the design needs to be careful. For example, the introduction of steric hindrance groups can reduce non-specific reactions of the probe with other reactive species, and the reactivity of the double bond to ONOO^−^ can be enhanced by altering the electronic environment of the double bond.

Despite the progress in the development of boronate- and ketoamide-based fluorescent probes for ONOO^−^, there is still a need to search for more chemoselective responsive groups. Aromatic group (e.g., hydroquinone and benzopyrylium/benzopyran)-containing probes usually contain one or more aromatic rings, offering good chemical stability and electronic properties for effectively reacting with ONOO^−^ [124]. The conjugated system of aromatic rings not only provides high stability but also enhances the fluorescence response, making it suitable for biological detection. However, the introduction of the relatively bulky aromatic groups to fluorescent probes might cause solubility issues as well as toxicity to cells and organisms.

## 5. Summary and Outlook

This review surveys the recent literature on fluorescent probes for the detection of ONOO^−^, with a focus on the commonly used reactive groups for ONOO^−^. We have also provided a detailed account of the design rationale, reaction mechanisms, and the sensing performances of the probes for in vitro and in vivo bioimaging of ONOO^−^. The majority of the probes are capable of ONOO^−^ sensing with high specificity and sensitivity, unveiling the diverse roles of ONOO^−^ in regulating disease-relevant pathways. The results obtained from those studies highlight the promise of detecting ONOO^−^ for the diagnosis of diseases as well as monitoring of therapeutics targeting ONOO^−^-related conditions.

Despite the significant progress made in ONOO^−^ sensing, several limitations hinder their clinical translation [125,126,127,128,129,130]: (1) The water insolubility of certain probes limits their utility in biological systems; (2) some probes undergo structural changes that lead to a blue shift in the emission wavelength, resulting in a poor tissue penetration, which is disadvantageous for real-time imaging in vivo; (3) probes with inadequate stability have a short half-life after entering the body, restricting their applicability for monitoring chronic diseases; (4) the majority of existing ONOO^−^ probes lack equipment with a targeting agent, which might increase the possibility of false-positive signals; and (5) for organs with physiological barriers, like the brain, probes need to undergo effective blood–brain barrier penetration to detect ONOO^−^ in these areas.

To overcome the issue of poor water solubility, probes can be structurally modified or coated with hydrophilic polymers to enhance their aqueous solubility. These modifications can, in turn, improve the biocompatibility and potential for clinical application of the probes. To overcome the limitation of poor tissue penetration, the development of fluorescent probes with longer wavelengths, including those emitting in the near-infrared region, is a promising approach. This can enhance the ability of the probes to obtain real-time in vivo imaging. For in vivo imaging, it is crucial that small-molecule probes have sufficient retention time in the body. The in vivo half-life of probes can be prolonged through the modification of targeting motifs or long-circulating materials. Furthermore, incorporating targeting agents can also help minimize false-positive signals, thereby improving the accuracy of disease diagnosis.

## Data Availability

Data are contained within the article.
